# Genome-wide transcriptome profiling and spatial expression analyses identify signals and switches of development in tapeworms

**DOI:** 10.1186/s13227-018-0110-5

**Published:** 2018-11-09

**Authors:** Peter D. Olson, Magdalena Zarowiecki, Katherine James, Andrew Baillie, Georgie Bartl, Phil Burchell, Azita Chellappoo, Francesca Jarero, Li Ying Tan, Nancy Holroyd, Matt Berriman

**Affiliations:** 1 Division of Parasites and Vectors, Department of Life Sciences, The Natural History Museum, Cromwell Road, London, SW7 5BD UK; 2 Parasite Genomics, Wellcome Trust Sanger Institute, Wellcome Trust Genome Campus, Hinxton, Cambridge, CB10 1SA UK

**Keywords:** *Hymenolepis*, Tapeworms, RNA-seq, Transcriptomics, Differential gene expression, Transcription factors, Signalling factors, Post-transcriptional regulators

## Abstract

**Background:**

Tapeworms are agents of neglected tropical diseases responsible for significant health problems and economic loss. They also exhibit adaptations to a parasitic lifestyle that confound comparisons of their development with other animals. Identifying the genetic factors regulating their complex ontogeny is essential to understanding unique aspects of their biology and for advancing novel therapeutics. Here we use RNA sequencing to identify up-regulated signalling components, transcription factors and post-transcriptional/translational regulators (genes of interest, GOI) in the transcriptomes of Larvae and different regions of segmented worms in the tapeworm *Hymenolepis microstoma* and combine this with spatial gene expression analyses of a selection of genes.

**Results:**

RNA-seq reads collectively mapped to 90% of the > 12,000 gene models in the *H. microstoma* v.2 genome assembly, demonstrating that the transcriptome profiles captured a high percentage of predicted genes. Contrasts made between the transcriptomes of Larvae and whole, adult worms, and between the Scolex-Neck, mature strobila and gravid strobila, resulted in 4.5–30% of the genes determined to be differentially expressed. Among these, we identified 190 unique GOI up-regulated in one or more contrasts, including a large range of zinc finger, homeobox and other transcription factors, components of Wnt, Notch, Hedgehog and TGF-β/BMP signalling, and post-transcriptional regulators (e.g. Boule, Pumilio). Heatmap clusterings based on overall expression and on select groups of genes representing ‘signals’ and ‘switches’ showed that expression in the Scolex-Neck region is more similar to that of Larvae than to the mature or gravid regions of the adult worm, which was further reflected in large overlap of up-regulated GOI.

**Conclusions:**

Spatial expression analyses in Larvae and adult worms corroborated inferences made from quantitative RNA-seq data and in most cases indicated consistency with canonical roles of the genes in other animals, including free-living flatworms. Recapitulation of developmental factors up-regulated during larval metamorphosis suggests that strobilar growth involves many of the same underlying gene regulatory networks despite the significant disparity in developmental outcomes. The majority of genes identified were investigated in tapeworms for the first time, setting the stage for advancing our understanding of developmental genetics in an important group of flatworm parasites.

**Electronic supplementary material:**

The online version of this article (10.1186/s13227-018-0110-5) contains supplementary material, which is available to authorized users.

## Background

Tapeworms are parasitic flatworms (Platyhelminthes) characterised by complex life cycles and a segmented, or strobilar, body plan, considered to be evolutionarily novel adaptations to parasitism [1–3]. As agents of neglected tropical diseases, they are estimated to be responsible for over 2.8 million disability-adjusted life years [4] and account for up to 30% of cases of epilepsy in regions of high endemicity [5]. Less acute, but more prevalent and widespread cestodiases caused by *Hymenolepis* species and other tapeworms contribute to further morbidity, particular of children, and frequently co-occur with other helminth infections [6]. The dwarf tapeworm, *H*. *nana*, is the most commonly reported cestode in humans [7] and has been shown to be the causative agent of proliferative, metastatic ‘tumours’ in immunosuppressed individuals, making its ubiquity especially important in areas where there is high prevalence of HIV AIDS [8, 9].

The genetic signals and switches that underpin the complex development of parasitic flatworms have just begun to be investigated [10–12] despite their diversity in form, ontogeny and potentially unique somatic stem cell systems [13–15] that are central to their life histories and the diseases they cause [16, 17]. In contrast, free-living planarian flatworms have been classical models in developmental biology for well over a century, and in the last two decades the availability of genomic resources [18, 19], functional methods [20–22] and a wide range of cell-type markers in *Schmidtea mediterranea* have made planarians a preeminent model system for investigating the biology of regeneration [23]. Their somatic stem cells [24, 25], called neoblasts, have been studied intensively [26], and neoblast-like proliferative cell compartments underpin the development of all flatworms [27]. Gene regulatory networks that pattern their axes during growth and regeneration have been elucidated [28], including the seminal discovery of canonical Wnt signalling as the basis for head/tail decision-making [29–31]. This rapidly growing canon of literature provides an important framework for comparative investigations of gene regulation in other flatworms and will help to ameliorate the historic gulf between the fields of development and parasitology [10].

More recently, genomic resources [32–35] and methods for investigating gene expression have been developed in trematode (fluke) and cestode (tapeworm) model systems, including the human bloodfluke *Schistosoma mansoni*, the fox tapeworm *Echinococcus multilocularis* and the mouse bile-duct tapeworm *Hymenolepis microstoma*. Somatic stem cells are central to the complex life cycles of flukes and tapeworms, and neoblast-like proliferative cell compartments have been characterised in both groups [14, 15, 36, 37]. New tissue and organ-specific markers have been developed for use with confocal microscopy [38, 39] as have colorimetric and fluorescent in situ hybridisation methods for examining spatial gene expression [15, 40]. Genomic resources and empirical tools are thus now available to facilitate the study of their developmental genetics, enabling more direct comparisons with free-living flatworms and more distantly related animal groups.

**Fig. 1:**
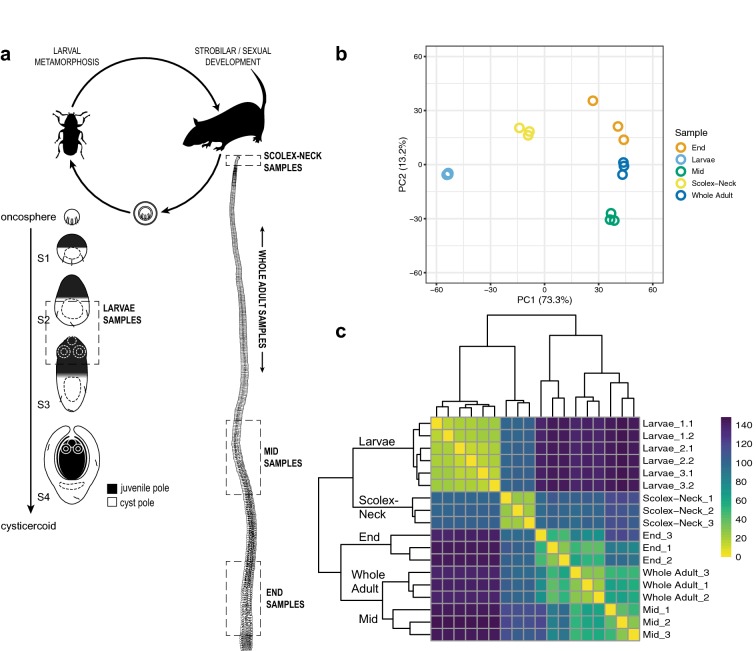
RNA-seq samples. **a** *Hymenolepis microstoma* life cycle illustrating the stages and regions sampled for transcriptome profiling (dashed boxes) described in Table 1 (whole worm illustration modified from [42]). **b** Principal component analysis showing clustering of sample replicates and relative variance among samples across PC1 and PC2. **c** Heatmap clustering of sample replicates showing hierarchical relationships based on overall expression similarity (n.b. each of the three larval samples was split and sequenced as two technical replicates). Note clustering of the Scolex-Neck samples with the Larvae samples rather than with the other adult samples

Few previous attempts have been made to identify the genetic factors regulating major developmental processes in tapeworms such as strobilation or larval metamorphosis. Prior to the advent of whole-genome sequencing methods, Bizarro et al. [41] employed empirical cDNA subtraction to address the process of strobilation, comparing differentially expressed genes in tetrathyridia and segmenting adults of *Mesocestoides corti*. Functional characterisation of the transcripts showed significant differences between the samples across the entire range of cellular processes, but pre-NGS methods only enabled a small number of factors to be identified. Here we use quantitative, whole-genome transcriptome profiling of the tapeworm *Hymenolepis microstoma* [42] to identify differentially expressed (DE) genes in Larvae and adults, and in the ‘Scolex-Neck’, ‘Mid’ and ‘End’ regions of the strobilar worm, broadly encompassing the major phases of development in the typical tapeworm life cycle (Fig. 1a). RNA-seq data were mapped to the *H. microstoma* genome (version 2), and details of this update to the published version 1 assembly [35] are presented here for the first time. Contrasts were made among the samples to identify DE signalling components (e.g. ligands and receptors) and transcription factors, often broadly referred to as ‘developmental control genes’ [43], as well as post-transcriptional/translational regulators, reasoning that these broad categories of genes are likely to include key regulators of the underlying developmental processes. Whole-mount in situ hybridisation (WMISH) in Larvae and adult worms was used to elucidate the spatial expression of a selection of these ‘genes of interest’ (GOI), enabling their expression to be linked to tissues, regions or organs in tapeworms for the first time. We present an overview of the sample transcriptome profiles and sets of DE gene models, and discuss factors examined by WMISH in detail, using their putative identities and spatial patterns together with published accounts of orthologs in planarians and/or other animals to infer potential roles of the gene products in tapeworms.

## Results and discussion

### The *Hymenolepis microstoma* v.2 genome

Our analyses utilised the unpublished v.2 release of the *H. microstoma* genome which is based on the inclusion of an additional Illumina HiSeq lane of data and re-assembly of the v.1 genome as described in Methods. In comparison with the v.1 genome published in Tsai et al. [35], the new assembly is larger (182 MB) and contains over 2,000 additional gene models supported by a combination of empirical RNA-seq data and bioinformatic predictions. All genome and gene model data are available online via WormBase ParaSite (WBP) (parasite.wormbase.org; [44, 45]) which includes a wide suite of bioinformatic tools for data access and interrogation, including the ability to dynamically generate gene trees and motif annotations using the most recent release of WBP and other major sequence databases.

### Sample overview

Biological characteristics of the samples in relation to development and metrics associated with their transcriptome profiles are given in Table 1, and the sample regions are illustrated graphically with respect to the *H. microstoma* life cycle in Fig. 1a. Mapping of the RNA-seq data to the genome resulted in 90% of the 12,371 gene models having normalised counts of one or more in at least one sample replicate, indicating that the transcriptome profiling captured the majority of predicted protein-coding genes. The overall percentage of models expressed was nearly identical among samples (82–83%), save the Scolex-Neck in which it was ~ 5% less (77%; Table 1). Full listings of raw and normalised counts are given in Additional file 1: Tables S1.2 and S1.3, respectively.

**Table 1:** RNA-seq sample descriptions and metrics

Sample	Replicates	Host/location	Description	Main developmental process	Specific developmental processes	No. of genes expressed (% total^a^)	Comparison	No. of up-regulated^b^ (% of expressed genes)	GOI^c^
Larvae	3 × apx. 550 pooled S2–S3 Larvae	Beetle (*Tribolium* spp.)/haemocoel	5-day old, mid-metamorphosis Larvae	Cysticercoid morphogenesis	Specification of primary body axes (AP/DV/LR) involving differentiation of juvenile worm and cyst-/tail-forming poles. Nascent nerve-muscle development in cyst and juvenile poles (e.g. rostellar apparatus, suckers)	10,257 (83%)	Larvae cf. Whole Adult	2266 (18%)	72
Whole Adult	3 × whole, gravid worms	Mouse (*Mus musculus*)/bile duct	Whole, gravid worms	Strobilar and sexual development	(encompassing all processes below)	10,139 (82%)	Whole Adult cf. Larvae	3087 (30%)	n/a
Scolex-Neck	1 × 13, 1 × 14, 1 × 43 pooled scolex + neck pieces (~ 0.5 cm each)		Scolex and ‘neck’ (including a variable number of nascent segments)	Proglottisation and strobilation	Formation of nascent segments and proglottids; cellular integration into existing muscle, nerve and excretory systems	9564 (77%)	Scolex-Neck cf. Mid/Scolex-Neck cf. End	1052 (11%)/812 (8%)	61/49
Mid	1 × 11, 1 × 12, 1 × 15 pooled pieces (~ 2.5 cm each)		Segments with mature proglottids	Sexual maturation	Gametogenesis and maturation of female system; fertilisation	10,170 (82%)	Mid cf. Scolex-Neck/Mid cf. End	2066 (20%)/457 (4.5%)	60/16
End	1 × 11, 1 × 12, 1 × 17 pooled pieces (~ 2.5 cm each)		Sub-terminal, gravid segments	Egg and embryo maturation	Embryogenesis; proglottid senescence	10,095 (82%)	End cf. Scolex-Neck/End cf. Mid	1440 (14%)/924 (9%)	22/18

^a^Of 12,371 gene models in the PRJEB124 release of the *Hymenolepis microstoma* genome (www: parasite.wormbase.org). Raw and normalised RNA-seq counts are listed in Additional file 1

^b^Differentially expressed gene models are listed in Additional file 2

^c^Genes of interest: signalling components, transcription factors and post-transcriptional/translational regulators (highlighted in Table S2.2-S2.5, Additional file 2)

Principal component analysis of the sample replicates (Fig. 1b) showed that they cluster tightly, save the End samples that showed a higher degree of dispersion. Eighty-six percent of the total variation was characterised by the first two principal components, with PC1 broadly separating the Larvae and Scolex-Neck samples from the Mid, End and Whole Adult samples, and PC2 separating the Mid and End samples from the others. The degree of similarity among replicates was higher than might have been expected, given that all samples were based on multiple individuals (save the Whole Adult samples; Table 1) and that there was inherent variability in both the degree of development among Larvae (which nevertheless showed the least dispersion) and in the ability to sample precisely the same regions of the strobila from different individuals. Heatmap analysis of sample-to-sample distances (Fig. 1c) showed that the Scolex-Neck samples were more similar in overall expression to the Larvae than to the other adult samples. Meanwhile, the Mid, End and Whole Adult samples formed a nested relationship in which the Whole Adult samples were closest to the Mid samples, suggesting that the profiles for Whole Adults were dominated by factors relating to sexual development, as would be expected given the proportion of the adult worm represented by the strobila.

**Fig. 2:**
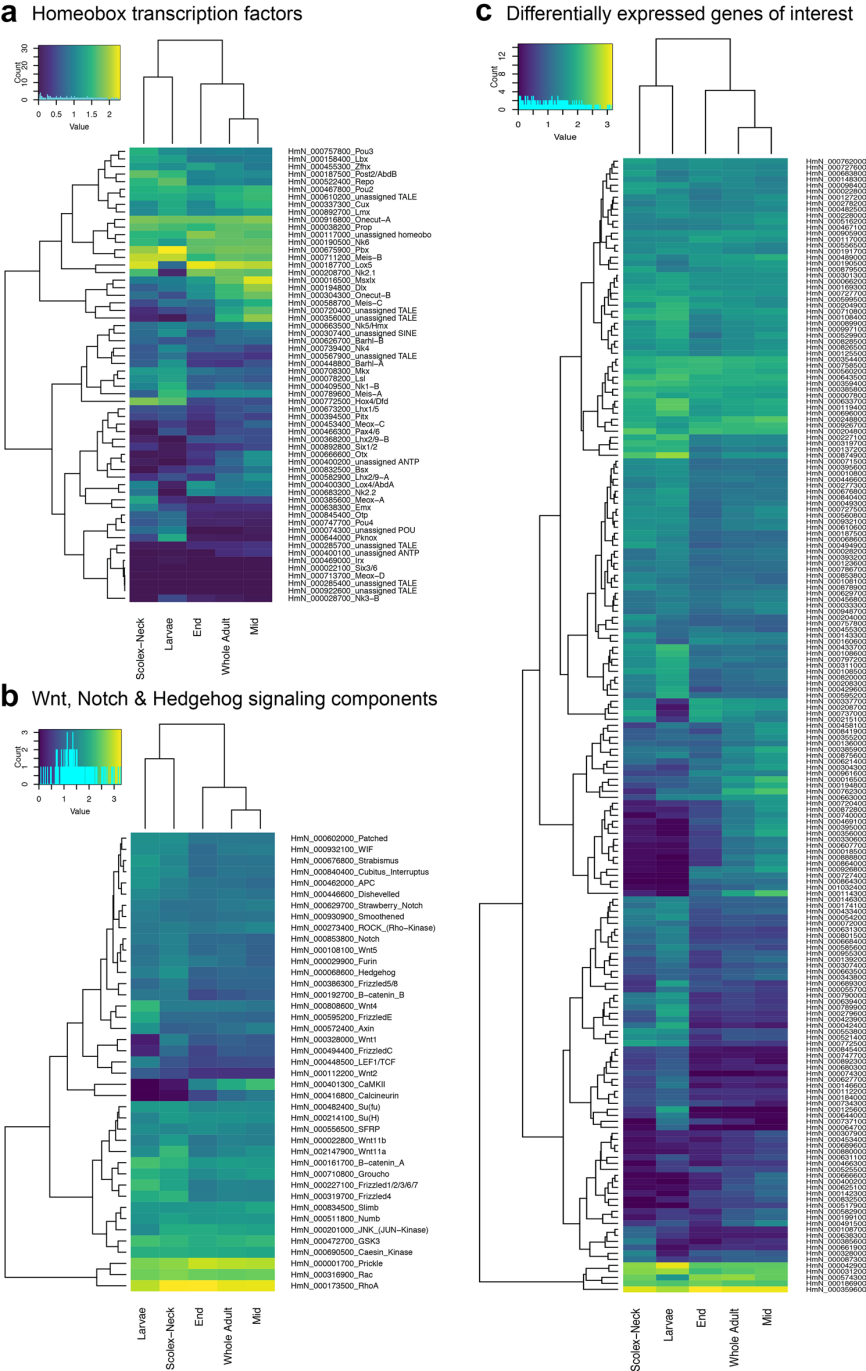
Heatmaps based on mean, normalised expression of selected factors representing ‘signals’ and ‘switches’. **a** All homeobox transcription factors [35]. **b** Wnt, Notch and Hedgehog signalling components [35, 63, 178]. **c** Differentially expressed genes of interest (Table 2, Additional file 4: Table S4; note that some of the factors in **a**, **b** were differentially expressed in one or more of the contrasts and are therefore also represented in **c**). All three suites of genes produce the same hierarchical clustering of the samples, congruent with that based on overall gene expression (Fig. 1c)

To examine similarities and differences among the samples in relation to our GOI, we constructed heatmaps based on normalised, mean expression values of three suites of genes: all homeobox transcription factors (Fig. 2a), components of Wnt, Notch and Hedgehog signalling pathways (Fig. 2b) and the GOI identified here (Fig. 2c), discussed below. In all cases, clustering of the samples produced the same branching pattern as the sample-to-sample comparisons based on all gene models. Moreover, hierarchical clustering united the Scolex-Neck and Larvae as sister groups, whereas independent branches would be more likely if their relationship was simply a reflection of their relative dissimilarity to the other samples. This relationship was further illustrated by large overlap in GOI DE in both the Larvae (cf. Whole Adults) and Scolex-Neck (cf. Mid and/or End) samples (Table 2; Additional file 1: Table S1), discussed below. We suggest that these groupings reflect the overarching similarities and differences in the underlying developmental processes represented by the samples (Table 1): mid-metamorphosis Larvae and the neck region of adult worms both exhibit extensive tissue re-modelling and patterning, whereas the Mid and End, and consequently, Whole Adult samples, exhibit growth predominately in relation to sexual and reproductive development.

**Table 2:** Up-regulated genes of interest

Contrast				Log2fold change (transformed mean expression Sample A–Sample B)						
Gene model	Type	Classification	Gene	Larvae cf. Whole Adult	Scolex-Neck cf. Mid	Scolex-Neck cf. End	Mid cf. Scolex-Neck	Mid cf. End	End cf. Scolex-Neck	End cf. Mid
**Larvae cf. Whole Adult**										
***HmN_000064700***	***TF***	***PRD homeobox***	***aris-like*** ^***b***^	7.4 (160–0)	–	–	–	–	–	–
HmN_000644000	TF	TALE homeobox	pknox^b^	6.9 (669–4)	–	–	–	–	–	–
HmN_000423900	SC	RTK signalling	FGFR1	3.5 (1125–96)	–	–	–	–	–	–
HmN_000737100	TF	ETS	elf2-like	3.3 (131–13)	–	–	–	–	–	–
HmN_000627700	TF	HMG-SOX	sox15	3.1 (331–37)	–	–	–	–	–	–
HmN_000595200	SC	Wnt signalling	fzdE^c^	2.7 (1875–282)	–	–	–	–	–	–
HmN_000680300	TF	Forkhead box	foxJ3-like	2.5 (134–22)	–	–	–	–	–	–
HmN_000892300	TF	LIM homeobox	lhm3/4	2.4 (57–10)	–	–	–	–	–	–
HmN_000174100	TF	T-box	tbx2-like	1.9 (902–249)	–	–	–	–	–	–
HmN_000112200	SC	Wnt signalling	wnt2^d^	1.8 (166–46)	–	–	–	–	–	–
HmN_000055700	SC	Notch signalling	delta-1	1.6 (500–168)	–	–	–	–	–	–
HmN_000089900	TF	E2F	e2f4-like	1.3 (1084–432)	–	–	–	–	–	–
HmN_000599500	PTR	RNA-binding protein	bruno4-like	1.1 (2284–1093)	–	–	–	–	–	–
HmN_000307400	TF	SINE homeobox	unassigned^b^	1.0 (196–96)	–	–	–	–	–	–
HmN_000446600	SC	Wnt signalling	dishevelled^b^	0.6 (1153–781)	–	–	–	–	–	–
**(Larvae cf. Whole Adult) AND (End cf. Scolex-Neck and Mid)**										
HmN_000689300	SC	RTK signalling	FGFR2	3.8 (1797–125)	–	–	–	–	2.0 (145–36)	1.7 (159–46)
HmN_000204900	TF	Forkhead box	foxO3/4	1.2 (3274–1441)	–	–	–	–	0.8 (1337–758)	1.2 (1464–634)
HmN_000191700	TF	CUT homeobox	cux/CASP-like	0.6 (1260–843)	–	–	–	–	0.9 (892–482)	1.2 (976–414)
**(Larvae cf. Whole Adult) AND (Scolex-Neck cf. Mid and/or End)**										
***HmN_000125600***	***TF***	***Forkhead box***	***foxQ2***	9.0 (866–0.5)	6.2 (22–0)	–	–	–	–	–
**HmN_000074300**	***TF***	***POU homeobox***	***pou-like*** ^***b***^	6.8 (100–0)	4.2 (44–2)	4.5 (36–0)	–	–	–	–
HmN_000042400	TF	PROX homeobox	prox1	5.8 (1035–17)	4.2 (127–7)	3.4 (105–7)	–	–	–	–
HmN_000772500	TF	ANTP-HOXL homeobox	hox4/dfd^e^	3.8 (1226–91)	4.7 (1424–53)	4.5 (1173–47)	–	–	–	–
***HmN_000845400***	***TF***	***PRD homeobox***	***otp*** ^***b***^	3.7 (157–12)	3.1 (89–10)	–	–	–	–	–
***HmN_000747700***	***TF***	***POU homeobox***	***pou4*** ^***b***^	3.3 (130–13)	3.4 (58–5)	–	–	–	–	–
HmN_000874900	TF	HMG-SOX	soxPF2	3.2 (8390–889)	2.1 (2621–632)	2.8 (2159–310)	–	–	–	–
HmN_000639400	SC	Notch signalling	delta-2	3.0 (1697–210)	3.1 (773–92)	3.0 (636–75)	–	–	–	–
HmN_000311000	TF	Forkhead box	foxD-like	2.6 (808–136)	1.4 (279–105)	–	–	–	–	–
***HmN_000553800***	***TF***	***basic Helix-Loop-Helix***	***myoD***	2.5 (920–158)	3.2 (909–103)	3.5 (749–65)	–	–	–	–
HmN_000227100	SC	Wnt signalling	fzd1/2/3/6/7^b^	2.4 (4783–883)	1.4 (1616–593)	1.6 (1331–439)	–	–	–	–
***HmN_000208300***	***TF***	***HMG-SOX***	***soxPF-1***	2.2 (1211–260)	–	1.0 (308–150)	–	–	–	–
HmN_000521400	TF	p54 family	p54-like	1.8 (475–134)	2.5 (519–91)	4.1 (427–23)	–	–	–	–
HmN_000797200	TF	p53 family	p53-like	1.8 (2287–640)	1.1 (714–343)	–	–	–	–	–
HmN_000676800	SC	Wnt signalling	strabismus-2^b^	1.6 (1810–586)	0.7 (731–442)	0.9 (602–326)	–	–	–	–
HmN_000319700	SC	Wnt signalling	fzd4/FzB^c^	1.6 (2942–994)	2.1 (3006–687)	2.2 (2476–530)	–	–	–	–
HmN_000494900	TF	HMG-SOX	soxPF-3	1.4 (482–185)	1.9 (680–182)	2.6 (560–91)	–	–	–	–
HmN_000049300	TF	Forkhead box	foxK2-like	1.3 (830–346)	–	1.3 (327–133)	–	–	–	–
HmN_000187500	TF	ANTP-HOXL homeobox	post2/abdB^e^	1.2 (843–355)	2.1 (1027–233)	2.1 (846–201)	–	–	–	–
HmN_000710800	SC	Notch signalling	groucho2^b^	1.0 (4350–2141)	–	0.9 (1895–1037)	–	–	–	–
HmN_000853800	SC	Notch signalling	notch1^b^	0.5 (1086–759)	1.4 (1143–438)	0.8 (942–537)	–	–	–	–
**Scolex-Neck cf. Mid and/or End**										
HmN_000638300	TF	ANTP-NKL homeobox	emx^b^	–	3.6 (97–7)	3.2 (80–7)	–	–	–	–
HmN_000385600	TF	ANTP-HOXL homeobox	meox-1^b^	–	3.3 (184–17)	5.3 (152–2)	–	–	–	–
HmN_000790000	TF	Lozenge	lz-2	–	2.7 (240–36)	3.0 (198–24)	–	–	–	–
***HmN_000137200***	***TF***	***bZIP***	***bZIP137200***	–	2.3 (1535–312)	–	–	–	–	–
HmN_000789900	TF	Lozenge	lz-1	–	2.2 (182–39)	1.8 (150–41)	–	–	–	–
HmN_000661900	SC	TGF-β/BMP signalling	bmp2-like	–	2.2 (87–19)	–	–	–	–	–
HmN_000146600	TF	ETS	Fli-1-like	–	2.2 (48–11)	–	–	–	–	–
HmN_000068600	SC	Hedgehog signalling	hedgehog^b^	–	1.8 (710–203)	1.8 (585–163)	–	–	–	–
HmN_000757800	TF	POU homeobox	pou3^b^	–	1.8 (576–167)	1.5 (475–168)	–	–	–	–
HmN_000328000	SC	Wnt signalling	wnt1^d^	–	1.8 (409–119)	1.4 (337–123)	–	–	–	–
HmN_000801500	TF	GATA	GATA2-like	–	1.5 (194–68)	–	–	–	–	–
HmN_000737000	TF	ETS	elf-1-like	–	1.5 (423–151)	–	–	–	–	–
HmN_000108100	SC	Wnt signalling	wnt5^d^	–	1.3 (304–120)	–	–	–	–	–
HmN_000098400	SC	TGF-β/BMP signalling	noggin-like	–	1.3 (658–266)	0.9 (542–280)	–	–	–	–
HmN_000022800	SC	Wnt signalling	wnt11b^d^	–	1.2 (877–373)	1.4 (723–267)	–	–	–	–
HmN_000010800	TF	Hippo signalling	TEF-5-like	–	1.2 (854–368)	–	–	–	–	–
HmN_000071500	TF	ETS	elf-like	–	1.2 (453–195)	–	–	–	–	–
HmN_000359400	SC	Wnt signalling	sfrp-like/SFL^c^	–	0.9 (2930–1523)	–	–	–	–	–
HmN_000683800	TF	MADS-box	mef2-like	–	0.9 (1220–656)	–	–	–	–	–
HmN_000204800	TF	CCAAT/EBP	c/ebp-like	–	0.9 (1321–725)	–	–	–	–	–
HmN_000762000	TF	Forkhead box	foxP1-like	–	0.8 (1182–657)	–	–	–	–	–
HmN_000395600	TF	Nuclear receptor	2DBD-like	–	0.8 (800–449)	–	–	–	–	–
HmN_000727700	SC	TGF-β/BMP signalling	smad4-like-2	–	0.8 (435–246)	–	–	–	–	–
HmN_000031200	PTR	RNA binding	PA2G4-like	–	0.5 (4610–3218)	–	–	–	–	–
HmN_000433400	TF	Wnt signalling	strabismus-1	–	–	2.2 (603–131)	–	–	–	–
***HmN_000204000***	***SC***	***TGF-β/BMP signalling***	***tgfb-like***	–	–	2.0 (532–129)	–	–	–	–
HmN_000878900	SC	TGF-β/BMP signalling	admp-like	–	–	1.3 (373–152)	–	–	–	–
HmN_000932100	SC	Wnt signalling	WIF^c^	–	–	1.2 (236–104)	–	–	–	–
HmN_000997100	PTR	DEAD-box helicase	unidentified	–	–	0.9 (791–413)	–	–	–	–
HmN_000125500	PTR	Pumilio	pum1	–	–	0.8 (1107–634)	–	–	–	–
**Mid cf. Scolex-Neck**										
HmN_000727400	TF	Forkhead box	foxA-like	–	–	–	8.0 (267–0.9)	–	7.4 (330–0.8)	
HmN_000114300	PTR	Piwi-like argonaute	group 4 argonaute^f^	–	–	–	7.2 (5463–31)	–	–	–
***HmN_000666600***	***TF***	***PRD homeobox***	***otx*** ^***b***^	–	–	–	7.2 (153–0.8)	2.6 (132–17)	–	–
HmN_000356000	TF	TALE homeobox	TALE-like^b^	–	–	–	7.1 (905–6)	–	–	–
***HmN_000762300***	***PTR***	***Boule/DAZL***	***boule2***	–	–	–	7.0 (3207–25)	2.9 (2783–225)	–	–
***HmN_000995400***		***ubiquitin ligase complex***	***zyg11-like***	–	–	–	7.0 (1384–11)	–	6.1 (733–9)	–
HmN_000832500	TF	ANTP-NKL homeobox	bsx^b^	–	–	–	6.8 (44–0)	–	–	–
HmN_000607700	SC	RTK signalling	aFGF-like-2	–	–	–	6.4 (372–4)	–	–	–
HmN_000466300	TF	PRD homeobox	pax4/6^b^	–	–	–	6.3 (50–0.3)	–	–	–
HmN_000018500	SC	RTK signalling	aFGF-like-2	–	–	–	6.2 (331–4)	–	–	–
HmN_000400200	TF	ANTP-HOXL homeobox	post2-like^e^	–	–	–	6.0 (155–3)	–	–	–
HmN_000720400	TF	TALE homeobox	TALE-like-2^b^	–	–	–	6.0 (470–7)	3.0 (407–38)	–	–
HmN_000625100	TF	RFX winged-helix DBD	rfx3-like	–	–	–	5.9 (268–4)	2.5 (233–32)	–	–
HmN_000469100	TF	TALE homeobox	meis-like	–	–	–	5.6 (507–9)	–	–	–
***HmN_000199100***	***TF***	***PRD homeobox***	***pax-like***	–	–	–	4.7 (141–6)	1.8 (122–33)	–	–
HmN_000194800	TF	ANTP-NKL homeobox	dlx^b^	–	–	–	4.6 (1422–59)	–	–	–
***HmN_000016500***	***TF***	***ANTP-NKL homeobox***	***msxlx*** ^***b***^	–	–	–	4.4 (1037–48)	–	–	–
HmN_000453400	TF	ANTP-HOXL homeobox	meox-3^b^	–	–	–	4.0 (27–1)	–	–	–
***HmN_000142300***	***TF***	***Forkhead box***	***foxC-like***	–	–	–	3.9 (144–9)	–	–	–
HmN_000582900	TF	LIM homeobox	lhx2/9^b^	–	–	–	3.6 (163–13)	2.9 (141–15)	–	–
HmN_000304300	TF	CUT homeobox	onecut	–	–	–	3.3 (1359–135)	–	–	–
HmN_000307900	PTR	DEAD-box helicase	ddx17-like	–	–	–	3.3 (75–7)	–	–	–
HmN_000525500	TF	DM domain sex-determining	dmrt-like	–	–	–	2.9 (114–15)	–	–	–
HmN_000961600	TF	PROX homeobox	prox-2	–	–	–	2.7 (460–69)	–	3.3 (589–57)	–
HmN_000385900	TF	E-box	E2-alpha-like	–	–	–	2.4 (1681–321)	–	–	–
HmN_000343800	TF	basic Helix-Loop-Helix	HES2^b^	–	–	–	2.4 (130–25)	–	–	–
HmN_000631100	TF	B cell factor family	ebf/coe-like	–	–	–	2.3 (135–27)	–	–	–
HmN_000621400	TF	C2H2 zinc finger homeobox	zfh-1	–	–	–	1.9 (795–216)	–	–	–
HmN_000875600	TF	Forkhead box	foxJ1-like	–	–	–	1.7 (1116–341)	2.3 (967–176)	–	–
HmN_000516200	PTR	Pumilio	pum2	–	–	–	1.4 (1969–757)	–	–	–
HmN_000467100	TF	X-box	nfx1-like	–	–	–	1.0 (1295–641)	–	–	–
HmN_000663500	TF	ANTP-NKL homeobox	nk5/hmx^b^	–	–	–	1.0 (1102–563)	–	–	–
HmN_000278200	TF	Basic leucine zipper	bZIP278200	–	–	–	0.9 (294–155)	–	–	–
HmN_000190500	TF	ANTP-NKL homeobox	nk6^b^	–	–	–	0.8 (944–541)	–	–	–
HmN_000629700	TF	Notch signalling	strawberry notch^b^	–	–	–	0.7 (1317–819)	–	–	–
**(Mid cf. End) AND (End cf. Scolex-Neck)**										
HmN_000458100		ubiquitin ligase complex	zyg-11-like	–	–	–	–	2.2 (793–164)	1.6 (150–47)	–
**End cf. Scolex-Neck and/or Mid**										
HmN_000663000	TF	HMG-Sox	sox5/6	–	–	–	–	–	3.1 (639–71)	–
HmN_000117000	TF	ANTP-NKL homeobox	barh-like^b^	–	–	–	–	–	1.6 (627–202)	1.1 (685–312)
HmN_000560200	TF	basic Helix-Loop-Helix	bigmax-like	–	–	–	–	–	1.4 (2059–781)	1.1 (2251–1019)
HmN_000337700	SC	Slit-Robo signalling	slit-like	–	–	–	–	–	1.3 (984–393)	–
HmN_000208700	TF	ANTP-NKL homeobox	nk2.1^b^	–	–	–	–	–	1.1 (1107–511)	0.8 (1210–684)
HmN_000354400	SC	Wnt signalling	GSK3-like	–	–	–	–	–	0.9 (1823–935)	–
HmN_000758500	PTR	Pumilio	pum3	–	–	–	–	–	0.8 (4341–2460)	0.8 (4742–2621)
HmN_000556500	SC	Wnt signalling	sfrp^c^	–	–	–	–	–	0.7 (751–449)	1.1 (823–391)
HmN_000215100	TF	Basic leucine zipper	bzip-574300	–	–	–	–	–	–	1.8 (493–132)
HmN_000727600	SC	TGF-β/BMP signalling	smad4-like-1	–	–	–	–	–	–	0.8 (775–429)

^a^Genes of interest: transcription factors (TF) (n.b. zinc fingers are listed separately in Table S3, Additional file 3), signalling components (SC) and post-transcriptional/translational regulators (PTR). Spatial expression patterns are discussed in the text for gene models shown in bold

^b^Gene identification based on Tsai et al. [35]

^c^Gene identification based on Koziol et al. [63]

^d^Gene identification based on Riddiford and Olson [178]

^e^Gene identification based on Olson [10]

^f^Gene identification based on Skinner et al. [13]

### Overview of differentially expressed genes

We considered a gene model to be differentially expressed if the difference between the mean, normalised sample counts was greater or less than zero at a confidence level of 1e-05, irrespective of the magnitude of difference. We ranked results by their log2fold change but did not impose an arbitrary cut-off to limit the number of DE genes taken into consideration. To identify up-regulated genes in the Larvae, we contrasted it with the Whole Adult sample, and not with the regional adult samples. To identify DE genes in adults, we made independent contrasts of each of the three regional samples with the other two, enabling DE gene models to be associated with different phases of strobilar and sexual development that are confounded by the Whole Adult sample. Full lists of DE gene models are given in Additional file 2: Tables S2.2–S2.5 for both sides of each contrast. For the reasons above, gene models up-regulated in the Whole Adult relative to Larvae are not considered further here but are included in Additional file 2: Table S2.2.

The percentage of gene models DE among the contrasts ranged from as low as 4.5% in the Mid sample (cf. End) to 30% in the Whole Adult sample (cf. Larvae; Table 1). The majority of the highest DE genes (e.g. top 20) among the contrasts were ‘novel’ proteins: predicted protein sequences lacking any significant similarity against the BLAST nr database (i.e. *e* values < 0.0001) or recognisable protein domains that were variously annotated as ‘n/a’ (no annotation), ‘expressed protein’, ‘expressed conserved protein’ (when supported by > 100 RNA-seq reads [35]), ‘hypothetical protein’ or ‘hypothetical transcript’. Putatively novel proteins make up just over half of all gene models (6455 of 12,371) and among contrasts ranged from 31 to 47% of the DE genes. In general, there were ~ 10% more novel gene models DE in contrasts involving the Whole Adult, Mid or End samples as compared to contrasts involving the Larvae or Scolex-Neck (Additional file 2: Table S2.1), suggesting that reproductive growth involves a larger percentage of putatively tapeworm-specific factors than either larval metamorphosis or strobilation.

### Gene Ontology

**Fig. 3:**
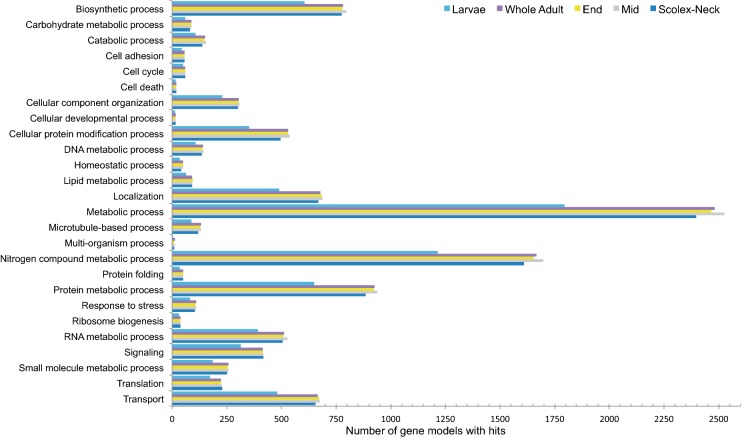
Comparison of Gene Ontology biological process annotations (slimmed) for the gene models across the samples. GO hits were counted only for models with mean, normalised expression levels ≥ 10. Note a generally smaller number of GO annotations in Larvae compared to the samples representing the adult worm

Gene Ontology (GO) [46, 47] terms associated with DE gene models were retrieved from WormBase ParaSite (parasite.wormbase.org; [44, 45]) and used to try and identify differences in term annotations among the samples (GO ‘hits’ to DE gene models are given in Additional file 2: Tables S2.2–S2.5). In total, 1962 unique terms were available, 6863 models had at least one annotation, and of these, 4254 models had a biological process annotation. When mapped to higher-level terms (i.e. GO slims), the pattern of annotation between the samples was highly consistent, with metabolic processes being the most represented in all cases (Fig. 3). Notably, despite the overall percentage of models expressed being nearly identical among samples, the Larvae had fewer GO slim annotated hits. Since these GO annotations are generated based on similarity to genes from well-studied organisms (i.e. humans and major model organisms; see Table 2 in [47]), this result suggests that the Larvae are expressing more genes that either have little similarity to other species and remain unannotated, or have annotations to terms that do not fall into the general GO slim categories. For the same reason, we suggest that the overall pattern (Fig. 3) is predominately one that reflects the ‘housekeeping’ state of the samples and is therefore highly similar, especially among adult samples.

We used GOseq [48] to look for biological process terms that had enriched representation among our sets of DE genes models using all models, and using only those with log2fold-change thresholds of 1.5 or 2.0. However, no biological process category was significant after multiple testing correction at any threshold. We therefore also used REVIGO [49] to reduce the number of terms by removing redundant, related terms. However, even using the ‘tiny’ option we found that terms were still too numerous for graphical overviews, and thus, we instead present lists of top-level terms for each contrast (Additional file 3: Tables S3.1–S3.7). In many cases, GO provided further support for the putative identities of the GOI, and for zinc finger proteins we used GO annotations to select gene models minimally including both ‘metal ion’ and ‘DNA-templated’ binding. Although in principle GO terms could be used to identify models representing major categories of genes, e.g. signalling or transcription, we found that hits against identified GOI were too inconsistent to be used reliably for this purpose. Given this result and the lack of enriched terms in our DE genes, we suggest that the application of this vocabulary to invertebrate, and especially lophotrochozoan, genomes is highly limited.

### Genes of interest

Among the DE gene models with annotations, we identified a total of 189 unique GOI up-regulated in one or more of the contrasts (listed in Table 2 and summarised in Additional file 2: Table S2.1; individual gene models highlighted in Additional file 2: Tables S2.2–S2.5). Forty percent of these were putative zinc finger transcription factors, the most abundant class of transcriptional regulators in animal genomes [50], and are compiled separately in Additional file 4: Table S4. The largest number of GOI (and DE genes in general) was identified in Larvae compared to the Whole Adult (Table 1; Additional file 2: Table S2.1), reflecting the coarseness of this contrast relative to those involving samples representing different regions of the adult worm which would be expected to share more similar constitutive (i.e. background) gene expression profiles, and thus a smaller percentage of genes DE. Not considering zinc fingers, 39 GOI were identified in Larvae, more than half of which were also found to be up-regulated in the Scolex-Neck sample when compared to the Mid and/or End samples. Conversely, little overlap in up-regulated GOI was found among other contrasts (Table 2).

Among the regional adult samples, contrasts involving the Scolex-Neck and Mid regions resulted in comparable numbers of GOI (60–61), whereas only ~ 1/3 as many were identified in the End sample (Table 1). This relative lack of up-regulated GOI could be explained by a loss of power due to the larger variation among replicates of the End sample (Fig. 1b). However, the overall number of DE gene models was comparable or greater in contrasts involving the End sample than in those involving other regions. Similarly, although there was a higher percentage (~ 10%) of novel DE genes in the End sample compared to the Scolex-Neck (and thus fewer annotated gene models from which GOI could be identified), this was equally true for the Mid sample. It therefore seems more likely that the relative lack of GOI identified from the End sample is due to developmental pathways up-regulated in the Mid and/or Scolex-Neck samples also operating in the End sample, and thus not found to be DE. For example, pathways regulating sexual development in the End are likely to be largely the same as those operating in the Mid sample, while pathways regulating some aspects of embryogenesis (e.g. axial patterning) may also be involved in strobilar growth in the Scolex-Neck sample. Thus, although developmental processes such as embryogenesis and senescence are uniquely represented by the End sample, the quantitative approach taken here was limited in its ability to identify associated factors.

### Differential expression during larval metamorphosis

Characteristically, the two most DE transcripts in Larvae were paralogs of the larval-specific tapeworm ‘antigen B-like’ protein [34] which were massively expressed (24,000 in Larvae cf. 0.5 in Whole Adults; Table S.2.2, Additional file 2). However, among DE GOI, more than half were also found to be up-regulated in the Scolex-Neck sample. Those with the highest fold change were the forkhead box gene *foxQ2* that was also up-regulated in the Scolex-Neck, followed by an *aristaless*-like homeobox which was DE only in Larvae. Other zinc finger, homeobox, forkhead box and high mobility group genes comprised the majority of transcription factors DE in Larvae only or in both the Larvae and Scolex-Neck samples. Among signalling components, two FGF receptors were identified, one of which was also up-regulated in the End sample, whereas components of Wnt and Notch pathways were enriched in both the Larvae and Scolex-Neck samples. A total of six Wnt components were identified in Larvae including a Wnt ligand and frizzled receptor DE only in Larvae, and two frizzleds up-regulated in both the Larvae and Scolex-Neck. Similarly, the Notch receptor *delta*-*1* was DE only in Larvae, whereas *delta*-*2* and the ligand *notch*-*1* were DE in both the Larvae and Scolex-Neck. Genes with putative roles in regulating stem cells included the post-translational regulator *bruno* [51], DE only in Larvae, and members of the *p53/54* transcription factor families [52], DE in both the Larvae and Scolex-Neck samples.

**Fig. 4:**
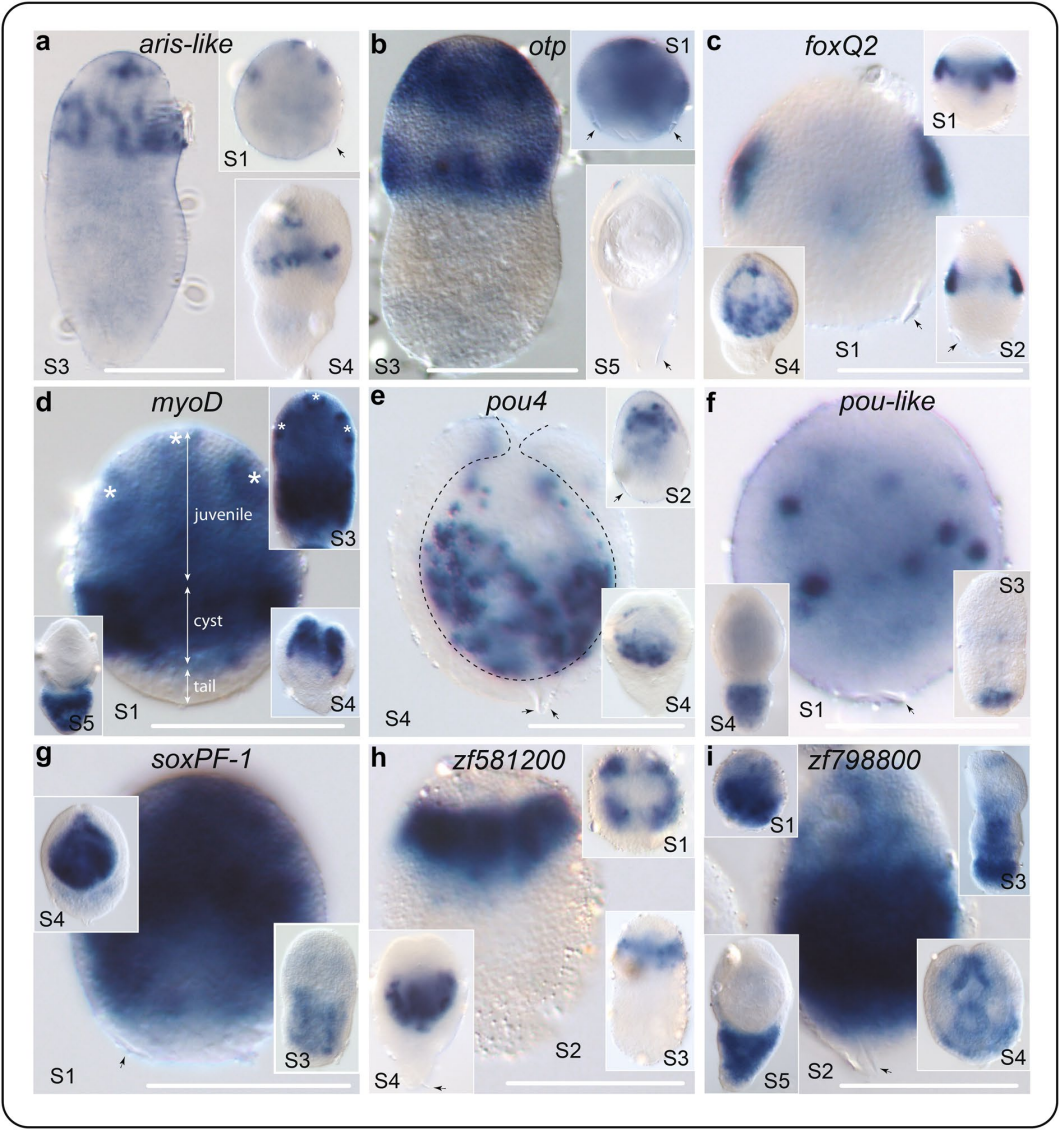
Spatial gene expression during larval metamorphosis. Larvae are staged as shown in Fig. 1a. Asterisks in **d** the nascent suckers and rostellar bulb, and arrows indicate the regions of the Larvae that give rise to the juvenile worm, cyst tissues and tail. Dotted line in **e** the boundary between the encysted juvenile worm and surrounding tissues. Arrows show oncospheral hooks where visible, indicating the larval posterior [63]. Gene models: *aris*-like (HmN_000064700), *foxQ2* (HmN_000125600), *myoD* (HmN_000553800), *otp* (HmN_000845400), *pou4* (HmN_000747700), *pou*-like (HmN_000074300), *soxPF*-1 (HmN_000208300), *zf581200* (HmN_000581200), *zf798800* (HmN_000798800). All scale bars 50 μm

Below we discuss spatial gene expression during larval metamorphosis of nine transcripts (Fig. 4), the majority of which were DE in both the Larvae and Scolex-Neck samples (Table 2). Included are two putative zinc finger transcription factors that were not part of those identified in Additional file 4: Table S4, but were previously identified in DE analyses based on an earlier version of the genome.

### *aristaless*

An *aristaless*-like Paired-class homeobox was highly DE in Larvae and had a transformed mean of zero in the Whole Adult (Table 2). It is expressed around the developing suckers and rostellum of the scolex, starting as one apical and two bilateral foci that expand and continue to be expressed post-encystment (Fig. 4a). At mid-metamorphosis, expression is broadly consistent with the spatial arrangement of the neurological connections that form together with the principal structures of the scolex (i.e. the apical rostellum and suckers) [39, 53], consistent with a role in the development of the central nervous system (CNS). In vertebrates, the *aristaless*-related homeobox gene *ARX* plays a pivotal role in neurogenesis and dysregulation is implicated in multiple neurological disorders [54], whereas it is involved in distal appendage formation in insects [55–57] and tentacle formation in *Hydra* [58]. Paired genes represent the second most diverse class of homeoboxes after ANTP [59], and many Paired-family genes are expressed in the nervous system of animals (e.g. *otp*, discussed below), including lophotrochozoans [60].

### *orthopedia*

A putatively CNS-related pattern of expression is also seen in an ortholog of the Paired-class homeobox *orthopedia* (*Otp*). *Hmic*-*otp* expression is diffuse in early-stage Larvae, but by mid-metamorphosis is restricted to the anterior hemisphere that gives rise to the juvenile worm. There it is expressed in large foci that overlap and encircle the Larvae in two ‘stripes’, one sub-apical and the other reaching the equator (Fig. 4b). *Otp* is a canonical regulator of brain development in animals [61], including planarians in which it is expressed in the outer branches of the brain during homoeostasis and in an apical patch in head blastemas during regeneration [62]. Although the expression pattern of *Hmic*-*otp* is more dispersed than that of *Hmic*-*aris*, it is broadly consistent with the areas where the cerebral ganglia and innervation of the suckers and rostellum.

### *foxQ2*

An ortholog of *foxQ2* is expressed in bilateral foci at the sides of the larva during the initial phase of metamorphosis and post-encystment becomes dispersed around the suckers (Fig. 4c). This is similar to the pattern seen in developing protoscoleces of the fox tapeworm *Echinococcus multilocularis* [63] and consistent with the patterning of the main branches of the CNS and innervation of the scolex. *FoxQ2* is a key regulator in the earliest stages of anterior patterning in animal embryos and in the development of the CNS [64–67]. As in tapeworms, *foxQ2* is not expressed apically in planarians, but in various parts of the nervous system, including photoreceptor neurons [68] and neural progenitor cells [69]. *FoxQ2* is also up-regulated in the Scolex-Neck sample, albeit at levels that are orders of magnitude less than in Larvae (Table 2), whereas no read mapped to this gene model in the Mid and End samples (Additional file 1: Table S1.2).

### *myoD*

Neurogenesis occurs in concert with muscle development, and in both the Larvae and Scolex-Neck samples we find strong up-regulation of a *myoD* ortholog, a universal regulator of myogenesis in animals [70]. WMISH shows clear spatial and temporal changes throughout metamorphosis, and the three regions of the nascent cysticercoid larva are readily demarcated by its expression (Fig. 4d). The anterior hemisphere shows diffuse expression throughout, whereas more concentrated expression makes visible the nascent suckers and rostellar bulb (marked by asterisks). The posterior hemisphere shows a strong band of expression in the cyst tissues around the ‘primary lacuna’ (i.e. larval cavity) in which thin sheets of muscle develop. Meanwhile, cells posterior to the cyst region that will subsequently form the ‘tail’ show no expression during early stages of metamorphosis. *Hmic*-*myoD* continues to be expressed strongly in the developing juvenile post-encystment (S4) and becomes restricted to the tail in the mature cysticercoid (Fig. 4d).

*MyoD* is a basic helix-loop-helix (bHLH) type transcription factor that has been described as a ‘master switch’ capable of inducing and orchestrating the differentiation of skeletal muscle cells in vertebrates [71]. Muscle development has been well described in planarians [72–74], and a *myoD* ortholog is expressed in both putative myogeneic progenitor cells [69] as well as fully differentiated myocytes [74]. More recently, Scimone et al. [75] showed that *Smed*-*myoD* does not play a generalised role in planarian muscle development, but is instead specific to the formation of a single muscle layer: the longitudinal muscles. Selectively inhibiting their formation via RNA interference revealed that the different muscle layers play distinct instructive roles during regeneration [75]. *MyoD* expression in *Hymenolepis* shows patterns consistent with the development of its larval muscle architecture, but further work is required to test whether its expression is specific to a particular layer.

### *pou4* and *pou*-*like*

Two POU-class homeoboxes show scattered, punctate expression. Spatial expression of *pou4* is restricted to the anterior, juvenile-forming half of the larva, with increased expression following encystment (Fig. 4e). *Pou*-*like* shows fewer foci more equatorially distributed during early metamorphosis, becoming diffuse and restricted to the posterior tail of the Larvae post-encystment (Fig. 4f). POU-class genes are found in all animals and are characterised by possession of separate POU and homeobox DNA-binding domains tethered by a variable linker region [76]. Parasitic flatworms possess orthologs of POU2, POU3, POU4 and POU6 family genes together with a single ‘orphan’ POU-like gene [35]. They have thus lost members of the POU1 family hypothesised to be present in the ancestor of the Lophotrochozoa, whereas the POU5 class is novel to vertebrates [77]. POU genes are involved extensively in nervous system development and in the regulation of stem cell pluripotency in vertebrates [76, 78, 79], and CNS-related expression has been shown in a range of lophotrochozoans, including planarians [80] and octopi [81]. POU genes are enriched in planarian neoblasts [82], and Scimone et al. [83] demonstrated the role of a *pou2/3* gene (putatively orthologous to *pou3* orthologs in parasitic flatworms [35]) in the development of the planarian’s protonephridial system. Restriction of *Hmic*-*pou4* to the areas of the developing scolex combined with conserved roles of POU genes in neurogenesis is consistent with its involvement in CNS development, like *aris, otp* and *foxQ2*. In contrast, the posterior, cyst-restricted expression of the ‘orphan’ *pou*-*lik*e gene makes it unlikely to be involved in either CNS or protonephridial development.

### *soxPF-1*

A Sox (SYR-like box) family transcription factor is expressed in a diffuse and dynamic fashion, appearing ubiquitous in S1 Larvae save the most posterior region, then seen in the nascent cyst tissues in S3, and finally restricted to the developing juvenile post-encystment (S4; Fig. 4g). *SoxPF*-*1* is one of three paralogs that are part of a parasitic flatworm-specific expansion of Sox genes, all of which show up-regulation in both the Larvae and Scolex-Neck samples (Table 2). In planarians, the *Schmidtea soxP1* gene and the presumably paralogous genes *Smed*-*soxP2* and *Smed*-*soxP3* are all expressed in neoblasts, but only *soxP1* is required for their long-term maintenance [84]. Sox genes are canonical regulators of metazoan stem cells capable of reprogramming differentiated cells [85, 86]. Spatial expression of *soxPF*-*1* in *Hymenolepis* may reflect cell proliferation during the different phases of metamorphosis, such as the formation of the cyst tissues and morphogenesis of the juvenile worm, and if so would be consistent with the gene product having a canonical role in regulating stem cells, as they comprise the only proliferative cell compartment in flatworms [27].

### *zf581200* and *zf798800*

Two unclassified, C2H2-type zinc finger transcription factors show strong expression in Larvae: *zf581200* is expressed in a quartet pattern in the anterior of the Larvae, prefiguring development of the suckers (Fig. 4h), while *zf798800* exhibits diffuse expression that becomes restricted posteriorly through the course of metamorphosis (Fig. 4i). In the present analyses, *zf581200* shows only 2 fragments mapped to Larvae (Additional file 1: Table S1.2), whereas gene model HmN_000798800 is not supported in the v.2 assembly, albeit our empirical data show that it represents a bona fide gene transcript. At least 37 types of specific binding domains characterise the zinc finger super-family, of which the C2H2 type is the most abundant [87, 88] and there are nearly 200 zinc finger genes in the *H. microstoma* genome [35]. It is hypothesised that lineage-specific expansion of transcription factors such as these plays a role in the changes in gene regulatory networks that produce unique traits in animals [88, 89], and thus, although almost all of the putative zinc fingers identified are unclassified (Additional file 4: Table S4), their spatial expression patterns would be nevertheless valuable to survey.

### Differential expression in the Scolex-Neck region

Differentially expressed GOI in the Scolex-Neck contrasts included a wide diversity of major types of transcription factors and more signalling components (18) than were found among the other contrasts. As previously discussed, nearly half of the GOI up-regulated in Scolex-Neck cf. Mid and/or End samples (35 of 77) were also up-regulated in the Larvae sample (Table 2 and Additional file 4: Table S4). Components of Wnt signalling included two Wnt ligands (*wnt1* and *wnt11b*) and repressors (*sfrp*-like and *wif*) and a paralog of *strabismus*, DE only in the Scolex-Neck, in addition to another *strabismus* paralog and two frizzled receptors DE in both the Scolex-Neck and Larvae. Notch signalling components, including orthologs of a Notch ligand and a Delta receptor, were also up-regulated in both the Scolex-Neck and Larvae samples, as noted above. Unlike the Larvae, however, DE GOI in the Scolex-Neck also included five components of TGF-β/BMP signalling, including *tgfb* and *bmp2*-like genes, an ortholog of Hedgehog, the primary ligand of the Hedgehog signalling pathway, and a *tef*-*5*-like transcript associated with Hippo signalling [90, 91]. One of three paralogs of the stem cell regulator Pumilio [92, 93] (*pum1*) is up-regulated relative to the End sample (but not to the Mid), whereas the remaining paralogs, *pum2* and *pum3*, are independently up-regulated in the Mid and End samples, respectively (Table 2).

Below we discuss spatial expression patterns in the Scolex-Neck and immature strobila of five genes:

### *snail*

**Fig. 5:**
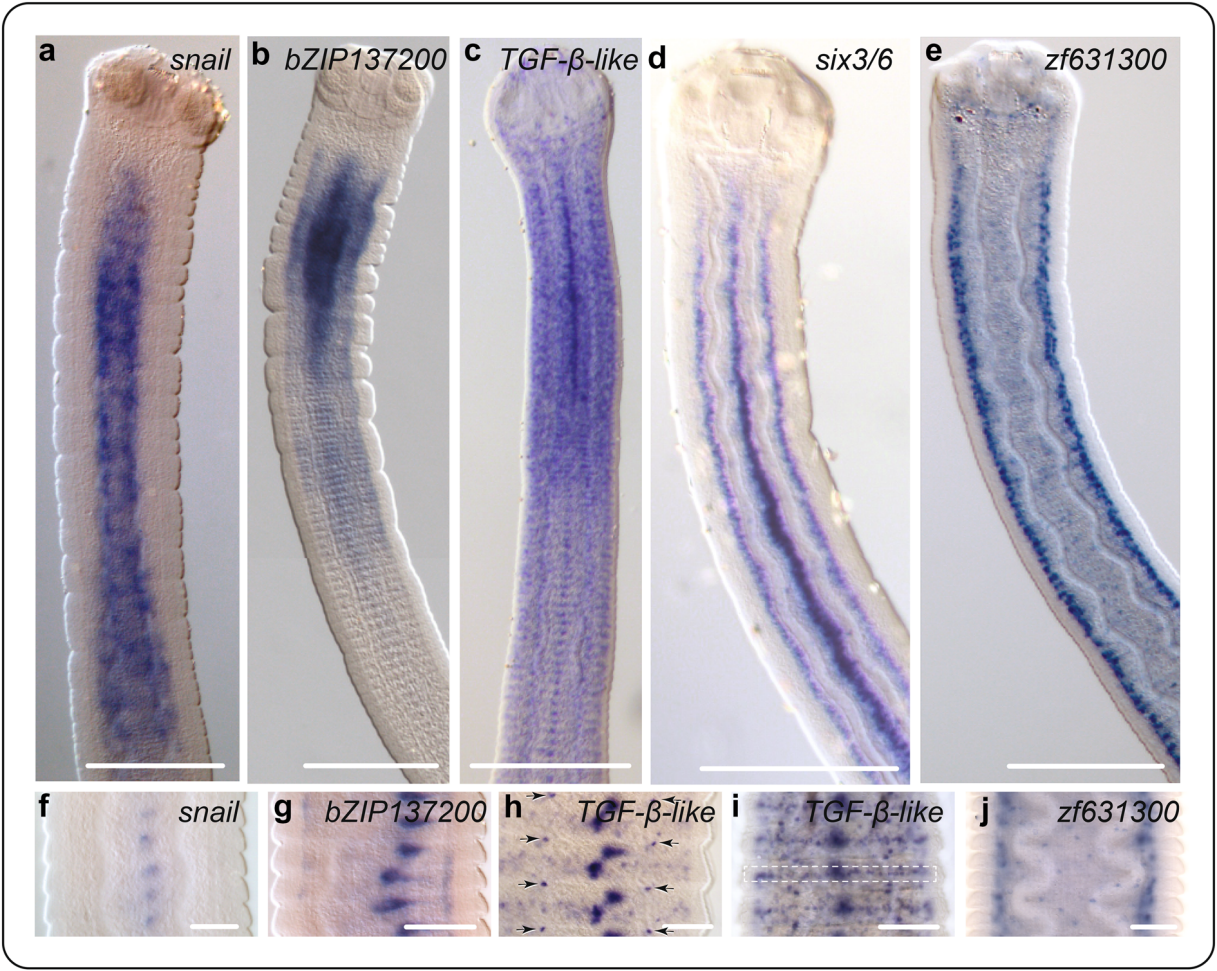
Spatial gene expression in the Scolex-Neck. **a**–**d** Expression in the scolex, neck and immature strobila. **f** Expression of *snail* in the genital primordia. **g** Expression of *bZIP137200* in the nascent seminal receptacle. **h**, **i** Expression of *tgfb*-*like* in immature segments. Arrows in **h** segmental, dorsoventrally paired foci, and dotted box in **i** punctate stripe of expression across the segments, both patterns consistent with the positions of segmentally repeated elements of the nervous system [39]. **j** Expression of *zf631300* showing punctate expression in the cortex. Gene models: *bZIP137200* (HmN_000137200), *six3/6* (HmN_000022100), *snail* (HmN_000348000), *tgfb*-like (HmN_000204000), *zf631300* (HmN_000631300). Scale bars 200 μm (**a**–**e**), 50 μm (**f**–**j**)

An ortholog of *snail* is expressed in a gradient around the medullary region and fades after the transition with the strobila (Fig. 5a). Further down the strobila, it is weakly expressed in the genital primordia (Fig. 5f). Here the gene model was not found to be DE but had been previously identified as such when the RNA-seq data were mapped to an earlier version of the genome assembly. *Snail* is a C2H2-type zinc finger transcription factor [94] encoding a protein that negatively regulates binding of *myoD* in progenitor myoblasts in mice, controlling their transcriptional state from one of proliferation to differentiation [95]. In *Schmidtea*, a direct ortholog of *Hmic*-*snail* is expressed together with *Smed*-*myoD* in a distinct population of muscle-related progenitor neoblasts [69]. Myogenesis in the neck is clearly required for the continual extension of the body by intercalation of new muscle cells. If *snail* plays a canonical role in tapeworms, then neoblast specialisation in planarians suggests that its expression pattern most likely represents a sub-population of germinative cells with a myogenic fate. Moreover, it would follow that what appears to be a lack of expression in the cortical (outer) region would be explained by migrating myogenic cells transitioning from a state of proliferation to one of differentiation [95].

### *bZIP137200*

An unclassified, basic-region leucine zipper (bZIP) transcription factor exhibits intense, diffuse expression in the neck that fades in a gradient at the transition with the strobila (Fig. 5b), as well as secondary foci of expression in the nascent seminal receptacle (Fig. 5g). Like bHLH transcription factors, bZIP proteins dimerise to form DNA-binding motifs and predate the origin of Metazoa [96]. The repertoire of bZIP class genes is largely unknown outside of major animal and plant models, especially in regard to lophotrochozoan taxa. However, a total of 29 bZIP genes were identified among the gene models of the Japanese oyster [97], including putative orthologs of many genes found in ecdysozoans and deuterostomes. The restricted expression of *bZIP137200* in the neck region and in the nascent reproductive organs points towards a role in a proliferative (= stem) cell compartment.

The patterns of both *snail* and *bZIP137200* appear to involve expression near the boundary of the medullary and cortical regions where there is a confluence of primary nerve elements, thick longitudinal muscle bundles and germinative cells [37, 39, 53]. Signalling between the nerve-muscle system and neoblasts is central to the developmental regulatory networks of planarians [98, 99], suggesting that this boundary in tapeworms represents a putative ‘signalling cylinder’ found not only the neck region, but extending the length of the strobila [12, 100]. As transcription factors act as up-stream or down-stream elements in signalling pathways, those DE in the Scolex-Neck are likely to be linked to developmental processes specific to the region.

### TGF-β-like

A TGF-β-like ortholog is expressed in multiple foci throughout the worm. In the neck, it is expressed in the genital primordia (misnamed the ‘primitive streak’ in tapeworms) and in a dense, punctate pattern that extends into the scolex (Fig. 5c). Beyond the neck a more regular, segmental pattern emerges that includes a central foci of expression representing the genital primorida that can be seen later to split antero-posteriorly (Fig. 5h) into the putative anlagen of the seminal vesicle/vagina and the ovary/vitellarium, or alternatively as a split between the male and female systems, as described in the rat tapeworm *Hymenolepis diminuta* by Sulgatowska [101]). Also visible is a quartet of dorsoventrally paired foci at the inter-segmental boundaries (Fig. 4h) corresponding in position to the junction of the medial longitudinal and traverse nerve cords, suggesting that these may act as centres of inter-segmental signalling. Finally, we observe a ‘stripe’ of expressing cells that circumscribes each segment at their equator (Fig. 5i).

TGF-β-like orthologs encode ligands of the highly conserved metazoan TGF-β/BMP signalling pathway and are involved in a wide range of morphogenic processes, often with pleiotropic effects [102, 103]. Ligands of TGF-β/BMP signalling fall into two broad superfamilies that stimulate different intracellular transducers (Smad proteins) resulting in the transcriptional regulation of different target genes. A direct ortholog of *Hmic*-*tgfb*-*like* in the bloodfluke *Schistosoma mansoni* (Add. File 1) was shown by Freitas et al. [104] to be expressed in the ovary and vitellaria of female worms (and sub-tegumentally in association with the oral and ventral suckers of males) and was deduced to have a role in embryogenesis. In *Hymenolepis*, we also see expression in the female system as well as many additional foci, suggesting multiple roles of TGF-β signalling throughout tapeworm development.

Using data from the *S. mansoni* genome [32] to represent flatworms, Kenny et al. [105] identified a total of five type I/II receptors and five Smads, but found only two TGF-β-type genes and no BMP-type gene. The large number of receptors relative to ligands suggested that some of the *S. mansoni* receptors may be responding to host ligands [106]. However, using the latest assemblies of the *S. mansoni* genome [33] on WBP we do find both TGF-β- and BMP-type orthologs in all major groups of parasitic flatworms, as well as in planarians. Moreover, a *bmp2*-like ligand is up-regulated in the Scolex-Neck (Table 2), but its spatial expression was not examined. The roles of TGF-β signalling via Smad2/3 in flatworms have not been identified, whereas the highly conserved role of BMP signalling in dorsoventral patterning of animals [107] has been well studied in planarians in which it is involved in both dorsoventral and midline patterning [108–113], and we would expect to find the same in other flatworms.

### *six3/6*

An ortholog of the sine oculus/Six homeobox family *six3/6* is expressed in three vertical ‘stripes’ in the neck and anterior strobila. Expression begins immediately following the scolex and runs along the dorsoventral midline just to the sides of the osmoregulatory canals and in the central ‘primitive streak’ (Fig. 5d). Posteriorly, expression becomes more punctate in the immature region of the strobila and then fades. *Six3/6* family genes, like *foxQ2*, are canonical regulators of anterior neural patterning during embryonic development [114, 115], and more generally, of early head patterning in animals [65, 116]. An *Echinococcus multilocularis six3/6* ortholog is expressed in protoscolices in the area of the rostellar nerve ring, the most apical region of the tapeworm CNS [63, 117]. In planarians, it is expressed in the outer cephalic branches of the brain [118] and in neural progenitor neoblasts [69]. Its spatial expression during strobilar growth in *Hymenolepis* is associated with the main longitudinal nerve cords, consistent with having a canonical role in the development and/or maintenance of the nervous system during strobilation and early stages of segment maturation, whereas expression in the genital primordia indicates an additional role in proglottid development. Like *snail* discussed above, *six3/6* was previously identified as DE in the Scolex-Neck and our empirical data corroborate this. However, only a few RNA-seq reads mapped against this gene model in the v.2 assembly (Additional file 1: Table S1.2).

### *zf631300*

*Zf631300* is an unclassified C2H2-type zinc finger DE in both the Larvae and Scolex-Neck (Additional file 4: Table S4) that in adults shows strong expression in association with the main elements of the CNS (Fig. 5e), as well as more dispersed, punctate expression in the tissues of the strobila (Fig. 5j). Expression associated with the nervous system is tightly clustered around the main longitudinal nerve cords and cerebrial ganglia in the scolex, appearing as individual foci (‘dots’) of expression surrounding the main nerve cords. One possibility is that this pattern represents expression by the nerves themselves which have been shown to be distinctly vesicular in tapeworms [53, 119, 120], suggesting that the CNS may act as a neurocrine organ [120, 121].

### Differential expression in the mature strobila

GOI in the Mid sample were dominated by transcription factors (Table 2; Additional file 3: Table S3), which made up 90% of the 59 GOI DE relative to the Scolex-Neck. Although zinc fingers were the most abundant type of transcription factor, as in all contrasts, homeoboxes made up a larger percentage of the total GOI in the Mid sample (28%) than in any other contrast, and included members of the HOXL, NKL, PRD, TALE, LIM, CUT and PROX families (Table 2; homeoboxes classified in S10.1 in [35]). The GOI with the highest fold change, however, was a forkhead box transcription factor, *foxA*-like. The only DE signalling components identified were two aFGF-like receptors, while two up-regulated transcription factors, *strawberry notch* and a member of the Hes (hairy and enhancer of split) family, are linked to Notch signalling [122]. Other GOI were post-transcriptional regulators that had among the highest transformed expression levels, including some whose products have canonical roles in stem cell regulation: a Piwi-like argonaute [13], the meiosis regulator Boule (discussed below), and one of three orthologs of Pumilio, noted above. Below we discuss the spatial expression of ten genes in the mature strobila, all of which proved to be associated with the female and/or male reproductive systems, and in multiple instances showed a second focus of expression consistent with the innervation of the genital pores.

### *boule2*

**Fig. 6:**
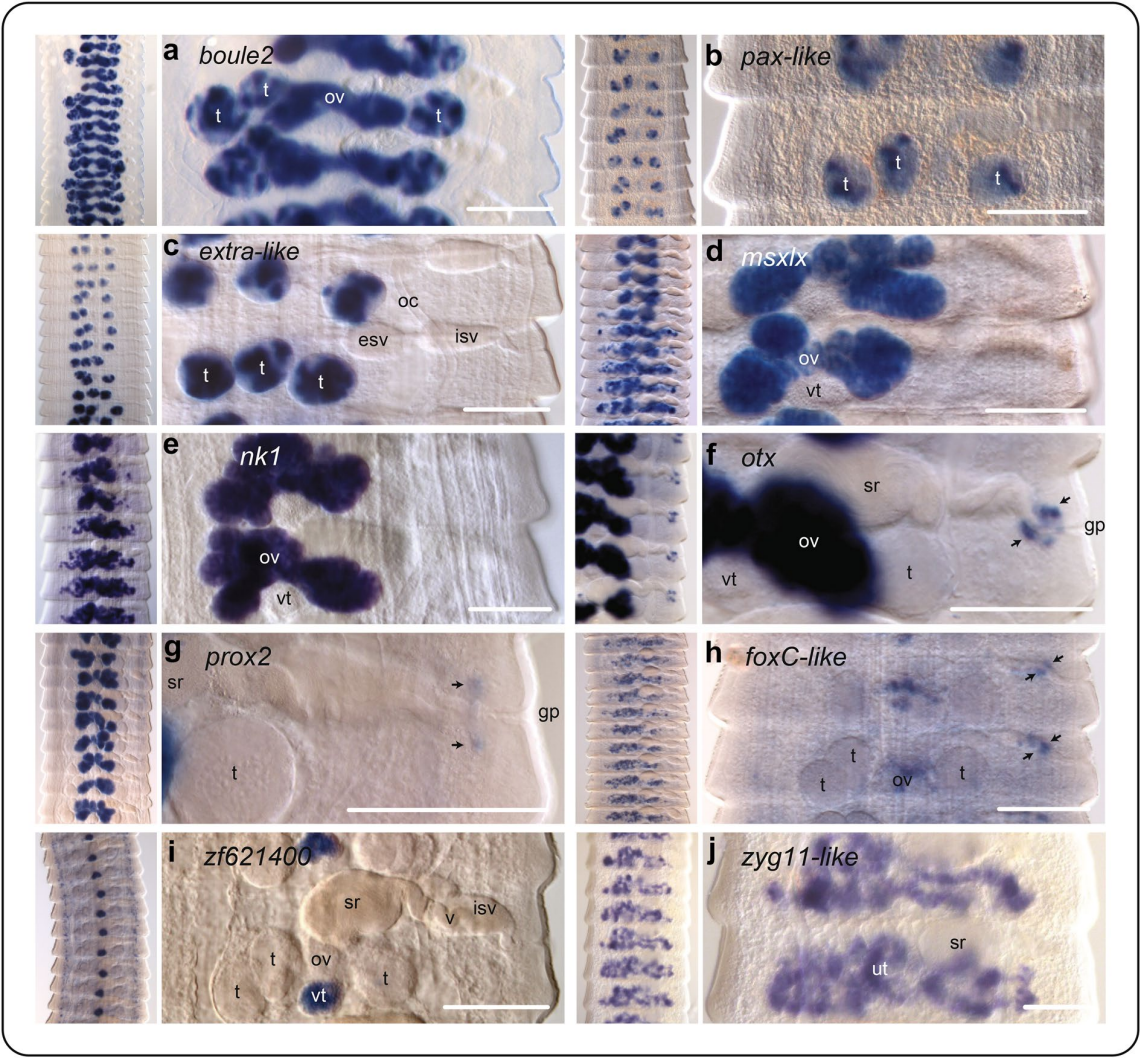
Spatial gene expression in the mature strobila. Arrows in **f**–**h** indicate expression foci around the genital pore. *esc* external seminal vesicle, *gp* genital pore, *esv* external seminal vesicle, *isv* internal seminal vesicle, *oc* osmoregulatory canal, *ov* ovary, *sr* seminal receptacle, *t* testes, *ut* uterus, *v* vagina, *vt* vitellarium. Gene models: *boule* (HmN_000762300), *extra*-*like* (HmN_000610200), *foxC*-*like* (HmN_000142300), *msxlx* (HmN_000016500), *nk1* (HmN_000601100), *otx* (HmN_000666600), *pax*-*like* (HmN_000199100), *prox2* (HmN_000961900), *zf621400* (HmN_000621400), *zyg11*-*like* (HmN_000995400). All scale bars 100 μm

An ortholog of the Boule/DAZL-family RNA regulator *boule* is strongly up-regulated in both the testes and ovaries (Fig. 6a). *Boule2* encodes an RNA-binding protein essential to germline development [51] and has been shown to regulate meiosis throughout the Cnidaria + Bilateria [123]. Boule expression in animals is generally male specific [123] but is associated with both the male and female germlines in flatworms, consistent with the expression foci seen in *H. microstoma*. The free-living flatworm *Macrostomum lignano* has three copies of *boule*, two of which are associated with the testes (one involved directly in spermiogenesis) and one with oogenesis in the ovaries [124]. In *Schmidtea*, there are also two paralogs, both of which are involved in spermatogenesis and oogenesis: Iyer et al. [125] showed that *Smed*-*boule1* has a canonical role in the regulation of meiosis, whereas *Smed*-*boule2* has a pre-meiotic role in the maintenance of early male germ cells, analogous but not homologous to the role played by DAZL genes in vertebrates. In the same year, Steiner et al. [126] showed that a *Schmidtea boule* ortholog (= *Smed*-*boule2* in [125]) is required for both male and female gamete production, but that whereas *Smed*-*boule2*(RNAi) results in sterility, it does not inhibit the production of egg capsules. Gene trees generated via WBP (not shown) indicate that a majority of parasitic flatworms have two paralogous Boule-DAZL-type genes that form separate clades, one of which includes *Smed-boule1* and *Smed-boule2*, whereas the other clade appears to be specific to neodermatans and includes the *Hmic*-*boule2* paralog analysed here.

### *pax*-*like* and *extra*-*like*

Testes-specific, regionalised expression was seen in a *pax*-*like* PRD-class gene (Fig. 6b) and an unclassified *extradenticle*-like TALE-class gene (Fig. 6c). Pax genes have been shown to regulate cell lineage specification and maintain progenitor cell populations through alternative splicing and gene activation/repression [127], and in mammals *pax7* expression is also specific to the male germline [128]. BLAST identification of the TALE-class gene returns *extradenticle* as the best match, but gene trees (not shown) suggest that it is one of at least eight unassignable genes that are part of a TALE-class expansion specific to parasitic flatworms [35]. Moreover, it is not orthologous to planarian *PBX/extradenticle* which is involved in anteroposterior (AP) polarity during regeneration by affecting Wnt signalling [129]. While there is scarcely information on the roles of these genes in invertebrates, spatial expression in *Hymenolepis* demonstrates their involvement in the male reproductive system.

### *msxlx* and *nk1*

Spatial expression of other up-regulated genes examined from the Mid sample was specific to the female system. Two ANTP-type homeoboxes, *msxlx* and *nk1*, are strongly up-regulated in the ovaries and continue to show expression in fertilised ova in the uterus (Fig. 6d, e). *Msx/msh* genes encode conserved proteins that act as transcriptional repressors involved in cell proliferation, differentiation and organogenesis during embryonic development [130]. Principal roles include differentiation of mesoderm into muscle and dorsoventral patterning of the neuroectoderm in concert with BMP signalling [131–133]. In Lophotrochozoa, roles in the differentiation of muscle and nerve precursors have been implicated in the polychaete *Platynereis* [134], and in dugesiid planarians *msh* genes have been shown to regulate neoblast proliferation and BMP signalling during head regeneration [135]. Ovarian and uterine expression of *msx* genes has been shown also in mice, including a direct role in the regulation of meiosis in the female germline [136, 137]. Tapeworms lack direct orthologs of Msx family genes [35] but do possess single copies of the closely related Msxlx family which has been lost in tunicates and vertebrates [138]. *Msxlx* expression in *Hymenolepis* is clearly related to the female system, and it would be informative to know whether it is also expressed in the ovaries of planarians. However, expression in planarians has only been examined in the context of regeneration [135].

*Hmic*-*nk1* is one of two paralogs found in tapeworms [35] and is a principal member of the NKL sub-class of ANTP-type homeoboxes that also includes the closely related Msx/Msxlx family genes [139, 140]. There is a large diversity of *nk*-*like* genes in animals which, similar to *msx* genes, play fundamental roles in differentiation, proliferation and apoptosis, often in relation to neurogenesis [134, 141] and muscle patterning [142], and an *nk1* gene has been shown to be essential for the formation of circular muscles in *Schmidtea* [75]. However, a recent study of the transcriptomes of the testes and ovaries of *Schistosoma mansoni* showed up-regulation of an *nk2* gene in the ovaries of paired females, but not in the testes of males or ovaries of un-paired females [143]. Thus together with the present study, results indicate a role of NK homeobox genes in the female reproductive system in both tapeworms and flukes.

### *otx* and *prox3*

In addition to the above NKL-type homeobox genes, the ovaries also express the PRD-class gene *otx* (Fig. 6f) and the PROS/Prox-class homeobox *prox/prospero* (Fig. 6g). Notably, *otx* and *prox3* also show another focus of expression around the genital pores, which are highly innervated [39, 119]. *Otx* is orthologous [35] to *Drosophila orthodenticle* (*otd*) and vertebrate *otx* genes, canonical regulators of brain development [144]. Planarians have two *otx* genes that are expressed in the brain [145, 146], in photoreceptor neurons [68, 147] and in CNS-related neoblast progenitors [69].

*Prospero/prox* genes are also broadly involved in nervous system development and in the regulation of neural progenitor cells [148–150] and in *Schmidtea* have been shown to be up-regulated in proliferating cells [84] including neural progenitor neoblasts [69]. *Prox2* is one of two paralogs in *Hymenolepis* and had few reads mapped against the v.2 genome (Additional file 1: Table S1.2) despite the strong expression seen in the ovaries, whereas *Hmic*-*prox1* was highly up-regulated in both the Larvae and Scolex-Neck samples (Table 2). A role of *Hmic*-*prox2* in the female germline of tapeworms is thus broadly consistent with stem cell-related roles in planarians. As both *prox* and *otx* genes are canonically associated with the nervous system, expression around the genital atrium is likely to be associated with its innervation. In contrast, *otx* has not been previously associated with the germline or reproductive tissues of animals. Simultaneous expression of these genes in the ovary and around the genital ducts points towards an intriguing link in gene regulation between these two parts of the reproductive system.

### *foxC*-like

A tapeworm-specific *foxC*-like forkhead box gene shows a similar, albeit weaker, pattern of expression in both the ovary and around the genital ducts (Fig. 6h). Weak expression is also observed in the nascent genital primoridia of the female system in immature segments, appearing as central dots (not shown). Gene trees produced via WBP (not shown) indicate *foxC*-*like* to be a divergent member of the family with direct flatworm orthologs identified only in the closely related species *H. nana* and *H. diminuta*. Thus although comparison with orthologs in other animals may be dubious, the *foxl2* gene has been shown to be central to ovarian differentiation and maintenance in mammals [151].

### *zf621400*

Of the transcripts found to be specific to the female system, only one showed expression in the vitellarium: an unclassified C2H2-type zinc finger. Vitellaria represent a division of the female system between the germarium (i.e. ovary; producing oocytes) and the vitellarium (producing vitellocytes) that is found in euneoophoran flatworms, which includes the parasitic flatworms and a subset of derived, free-living groups that includes planarians (i.e. Tricladia) [152, 153]. Ectolecithal eggs are produced in which fertilised oocytes are packaged with varying numbers of vitellocytes that provide both yolk and proteins for the production of the egg shell [154]. Typical of cyclophyllidean tapeworms, *H. microstoma* has a single, compact vitellarium situated centrally, below the bi-lobed ovary [42]. *Hmic*-*zf621400* expression is seen throughout the developing and mature vitellaria in the strobila (Fig. 6i) and in individual cells (putatively vitellocytes) in the uterus, showing that it continues to be expressed during embryogenesis (Additional file 5: Figure S1). However, in addition to the vitellaria, expression is also observed in somatic cells and in the genital primordia of immature segments (Additional file 5: Figure S1). Hence, it may be that this transcription factor had a more general role in flatworms and secondarily evolved a role in vitellogenesis in the Euneophora. A molecular marker for tapeworm vitellaria has not been reported previously, and thus, the gene may find utility for this purpose. A large number of vitellaria- and/or vitellocyte-specific genes were identified in blood flukes through a similar combination of RNA-seq and WMISH [155], and many of these may prove to be useful markers in other parasitic flatworms and other euneophoran groups.

### *zyg11-like*

A *zyg11*-*like* gene is up-regulated in the Mid and End samples (cf. Scolex-Neck) and was one of a small number of tapeworm orthologs identified among a list of candidate senescence-related genes in *C. elegans* (unpub. data). In *Hymenolepis*, it shows diffuse expression throughout the uterus, revealing the anastomosing structure of the organ (Fig. 6j). *Zyg11* encodes a member of a large protein family characterised by leucine-rich repeats and is involved in several functions in *C. elegans* including meiotic progression and AP polarity during embryogenesis [156, 157]. Functions of the gene family outside of *C. elegans* are not known. *Hmic*-*zyg11*-*like* is one of 12 paralogs in the *H. microstoma* genome, and phylogenetic analysis via WBP (not shown) indicates that it is part of a large expansion of this gene family in parasitic flatworms. Its spatial expression suggests it could be a marker for the uterus.

### Differential expression in the gravid strobila

A comparatively small number of DE GOI were identified in the End sample (Table 2) as discussed previously, and none showed high log2fold-change values. In addition to five putative zinc fingers, there was a small number of homeoboxes, high mobility group and basic leucine zipper transcription factors identified (Table 2). Among signalling components was an FGF receptor also up-regulated in Larvae (discussed above) and a slit-like protein sequence that was the only example among GOI of the Slit/Robo pathway which is canonically involved in axon repulsion during neural development [158]. A paralog of the *smad*-like factor DE in the Mid sample was identified as were components of Wnt signalling, including the Wnt antagonist *sfrp* which is involved in establishing primary AP polarity in animals [159] and in tapeworms is expressed at the site(s) of scolex formation in developing Larvae [63]. A paralog of the stem cell regulator *pumillio* (cf. Mid sample) was highly expressed, albeit only around twice the level of its expression in the Mid and Scolex-Neck samples (log2fold change of 0.8; Table 2). No gene was examined by WMISH in the End sample.

## Conclusions

Through genome-wide transcriptome profiling we have identified genes DE in association with the major stages of the *H. microstoma* life cycle, revealing candidate regulators of the underlying developmental processes. Spatial analyses corroborate quantitative RNA-seq data and provide insights into the putative roles of the genes by linking their expression to organs, tissues and cells in tapeworms for the first time. The overarching picture shows that their complex development involves factors with canonical roles in axial specification, neurogenesis, myogenesis and stem cell regulation and that their underlying developmental ‘toolkit’ therefore resembles that of free-living flatworms and metazoan animals in general. The striking similarity in transcriptome profiles of the Scolex-Neck region and Larvae, both in general and specifically in relation to genes known to be involved in animal development, suggests that strobilation in adults involves recapitulation of many of the same gene regulatory networks employed during larval metamorphosis. In contrast, reproductive development involves a larger percentage of up-regulated genes that lack clear orthologs in other animal groups.

Our work represents a course-grained survey of differential gene expression in tapeworms and a finer-grained approach including more time points during larval metamorphosis and smaller divisions of the adult body would reveal additional DE genes and provide a more refined overview of their temporal expression dynamics. Broad surveys of this type are especially critical in new model organisms and have been integral to elucidating gene regulatory networks in planarians [21]. Recent addition of long-read and optical mapping data has now improved *H. microstoma* genome assembly to the level of full chromosomes (unpub. data), which will make it among, if not the, most complete genome of a lophotrochozoan, and will stabilise gene model estimates. The development of such genomic resources for parasitic flatworms and their curation via WormBase ParaSite [45] has accelerated our ability to investigate their developmental genetics. Moreover, increased representation of species through the 50 Helminth Genomes Project [160] means that we can make more comprehensive investigations of the diversity and interrelationships of developmentally related genes to determine their degree of taxonomic restriction (i.e. ‘orphan-ness’) [161, 162] and to identify where direct orthologs exist between free-living and parasitic species.

Understanding the somatic stem cell systems of parasitic flatworms is essential to investigations of all aspects of their growth and differentiation. Germinative cells of tapeworms and neoblast-like cells of blood flukes have been a focus of recent studies that demonstrate similarities with planarians as well as potentially significant differences in their underlying regulation [14, 15, 36, 37, 100, 163, 164]. Transcriptional profiling of their proliferative cell compartments will allow us to determine the full extent to which regulation differs from the well-characterised neoblast system of planarians, and we expect that single-cell profiling will reveal sub-populations of partly differentiated progenitor stem cells, as recently characterised in *Schmidtea mediterranea* [69, 165]. Such studies would provide new candidate targets for chemotherapy as well as an abundance of cell-specific markers that will enable expressed genes to be associated with different cellular compartments.

## Methods

### Animal cultures and samples

The Nottingham strain [42] of the mouse bile-duct tapeworm, *Hymenolepis microstoma*, was maintained in vivo using flour beetles (*Tribolium confusum* and *T. castaneum*) and inbred strains of laboratory mice (*Mus musculus*) in accordance with project licence PPL70/8684 issued by the UK Home Office to PDO. To produce larval samples representing the approximate mid-point in the metamorphosis from oncosphere to cysticercoid, beetles were starved for five days and then exposed to macerated, gravid proglottids of *H. microstoma* for ~ 6 h. Gravid tissues were removed, and the beetles allowed to feed on flour ad libitum. Beetles were dissected five days post-exposure to eggs and the Larvae collected from the haemocoel and transferred live into RNAlater (Qiagen). Morphologically, most Larvae were elongated and well differentiated at both poles, equating to stage 3 following Voge [166]. However, individual variation in developmental rates meant that some Larvae were closer to ‘stages’ 2 or 4. Approximately 550 individuals were combined for each of three replicate larval samples and stored in RNAlater at − 80 °C.

For the Whole Adult samples, fully grown, gravid worms > 1 month old were dissected from the bile ducts of mice into vertebrate saline (0.85% w/v NaCl), rinsed and preserved live in RNAlater before storing at − 80 °C. Three entire worms were used as replicates for the Whole Adult samples.

For the regional samples of the adult worm, whole worms were removed from mice and swirled in near boiling saline to extend and kill the worms. They were then straightened in a petri dish while immersed in RNAlater, worms of similar length aligned, and comparable regions of the strobili cut from multiple worms and pooled. The Scolex-Neck samples included the scolex, neck and some number of nascent segments (total sample length apx. 0.5 cm from apex) and consisted of tissues combined from 13 to 43 individuals/replicate. The Mid samples consisted of ~ 2.5 cm lengths of tissue representing not the actual mid-point of the strobila, but a position roughly 2/3 of the length from the apex where both the male and female systems are mature (Fig. 1a) and were combined from 11 to 17 individuals/replicate. The End samples consisted of ~ 2.5 cm lengths of gravid, sub-terminal tissues combined from 11 to 14 individuals/replicate.

For in situ hybridisation, adult tapeworms were harvested from mouse bile ducts into saline and swirled for ~ 2 s in near boiling saline to extend and kill the worms as above. They were then fixed in fresh, cold 4% w/v paraformaldehyde (PFA) in phosphate-buffered saline (PBS) overnight at 4 °C before being transferred to PBSAT (PBS and 0.1% v/v Tween 20) or dehydrated in a graded series of ethanol and PBS and stored at 4 °C. Whole worms were rehydrated in PBSAT and cut into 2–4 cm sections prior to WMISH. Larval worms were produced as described above, and developmental series of Larvae were generated by sub-sampling infected beetles on days 3, 4, 5, 6 and 7 post-exposure to eggs and then fixing them directly in RNAlater. Prior to processing, the Larvae were visually sorted into ‘stages’ (Fig. 1a) and 5–10 individuals of each stage combined into individual tubes to be processed simultaneously. Further details on the culturing of *H. microstoma* are available at www.olsonlab.com.

### Transcriptome sequencing

Total RNA was prepared by washing each sample in ice-cold PBS before being mechanically homogenised in Trizol (Invitrogen) and extracted with 24:1 chloroform/isoamyl alcohol. Phase separation was carried out by centrifugation at 4 °C. 0.5 volumes isopropanol, and 4 µl of glycogen (5 mg/ml) was added to the aqueous phase, and total RNA was precipitated at − 80 °C for 1 h. RNA was pelleted, washed with fresh 75% v/v ethanol, re-suspended in nuclease-free water and quality-checked and quantified using an Agilent Bioanalyzer 2100. Libraries for transcriptome sequencing were prepared using the TruSeq kit (Illumina) with polyadenylated mRNA selected using oligo-dT Dynabeads. After cDNA synthesis, adaptors were ligated and libraries were amplified by PCR using Kapa HiFi DNA polymerase (Kapa Biosystems). Amplified templates were purified with AMPure SPRI beads, quantified using a Bioanalyzer (Agilent) and pooled. From pooled libraries, 300- to 400-bp fragments were selected using the Caliper system. After adaptor ligation, individual libraries made with the Illumina mRNA-seq kit were size-selected using the Caliper system before PCR amplification followed by AMPure SPRI bead clean up and removal of adaptors with a second Caliper run. Kapa Illumina SYBR Fast qPCR kit was used to quantify the Illumina mRNA-seq libraries before pooling. Libraries were sequenced using v4 Cluster Generation and v5 Sequence-by-Synthesis kits, according to the manufacturer’s protocols (Illumina), to produce paired 76- or 100-base reads. Primary analysis of raw data generated by the Illumina HiSeq genome sequencer was performed with the RTA v.1.8 analysis pipeline. Accession numbers of the generated sample data are given in Additional file 1: Table S1.1.

### *Hymenolepis microstoma* v.2 genome assembly

Analyses were based on the latest publicly available version of the *H. microstoma* genome and gene models (v.2) curated on WormBase ParaSite (parasite.wormbase.org) [45] under accession PRJEB124. This represents an unpublished update to the v.1 assembly published in [35] and has not been previously described. Version 2 is based on the incorporation of an additional lane of Illumina HiSeq data from the library ENA002564 (https://www.ebi.ac.uk/arrayexpress/) and re-assembly of the genome as follows. The v.1 genome was quality-checked using REAPR v.1.0.15 [167]. Low-confidence scaffolds were broken, and all sequence data were mapped back to the genome. Unmapped reads were assembled using Velvet v.1.2.09 with a kmer size of 57 [168]. Resulting contigs longer than 500 bp were merged with the REAPR-corrected genome and scaffolded using three iterations of SSPACE v.1.1 [169]. Gaps were filled using several increasingly permissive iterations of GapFiller v.1.11 until saturation was achieved [170]. The process of breaking and re-joining was repeated until only marginal returns could be achieved. Contigs shorter than 500 bp were excluded and consensus bases corrected using iCORN for three iterations. Using PROmer from the MUMmer package v.3.23 [171], it was observed that the *H. microstoma* genome is largely co-linear with that of the fox tapeworm *Echinococcus multilocularis*. The *H. microstoma* genome was thus ordered following orthologs to *E. multilocularis* using ABACAS v.2 [172]. As scaffolds were joined on orthology evidence, the resulting gene order may be incorrect if it has changed during evolution, but it represents our current best hypothesis of the interrelationships among contigs. The resulting v.2 assembly is 16% larger than v.1 and includes longer scaffolds and contigs.

Gene models were transferred from version 1 to 2 (7981 models transferred out of 10,149). As v.2 is larger than v.1, new gene models were predicted using Augustus v2.5.5 [173] with and without RNA-seq support. Only unique gene models not overlapping with a previously identified model were retained and in total, 2222 new gene models (of 12,368 in total) were included in the current set. Gene models were annotated using a custom pipeline as described in section S5 of [35]. In brief, annotations were made from the product calls of the top ten matches in the GenBank nr database where they had *e* values < 0.0001. For predicted proteins lacking such similarity to known sequences, annotation was made from any domains identified in the protein using InterProScan. Remaining proteins lacking domain predictions were annotated as novel as described in Results and Discussion. All genome data and annotations are available via WormBase ParaSite [45] at parasite.wormbase.org.

### Differential expression analyses

Paired-end RNA-seq reads from a total of 18 technical and sample replicates (Additional file 1: Table S1.1) were mapped to the genome using TopHat v.1.4.1 [174] and FPKM values (fragments per kilobase per million mapped reads) calculated for each gene model using the Cuffdiff programme included in the Cufflinks package (v.2.0.2; default parameters plus –u and –b options [174]). Raw and normalised mapped reads per gene model and sample replicate are given in Additional file 1: Tables S1.2 and S1.3, respectively. Differentially expressed genes were determined using DESeq2 v.1.14 [175]. Comparisons were made between the Larvae and Whole Adult samples and among each of the three regional samples (i.e. Scolex-Neck cf. Mid, Scolex-Neck cf. End, and Mid cf. End) as described in Results and Discussion. Raw counts per replicate were used as input, and the two technical replicates for each of the Larvae samples were combined using the collapseReplicate function. DESeq2-transformed and DESeq2-adjusted gene model expression levels between samples differing from zero at a confidence level of 0.00001 were considered differentially expressed and were ranked by their log2fold change. Complete lists of up- and down-regulated gene models for each contrast are given in Additional file 2: Tables S2.1–S2.5, and the intersects of up-regulated genes for each of the regional samples compared with the other two are given in Additional file 2: Tables S2.6–S2.8.

To investigate overall similarities/differences among the samples and replicates, we plotted the first two principal components of the libraries using the dists and plotPCA functions (Fig. 1b) and constructed a heatmap of the sample-to-sample distances using the pheatmap function (Fig. 1c). In addition, we constructed heatmaps based on normalised expression means of three suites of gene models: (1) all homeobox transcription factors (Fig. 2a); (2) all components of Wnt, Notch and Hedgehog signalling pathways (Fig. 2b), and (3) all unique GOI identified herein. Homeoboxes and signalling components are identified in Supplementary Tables S10.1 and S10.2, respectively, in [35], and GOI identified here are listed in Table 2 and Additional file 4: Table S4.

### Gene Ontology analyses

Gene Ontology [176] terms associated with the gene models were retrieved from WBP using the Biomart function and are included in Additional file 2: Tables S2.2–S2.5 and summarised in Additional file 3: Tables S3.1–S3.7. Biological process terms were mapped to GO slims using bespoke java code and GO version 2018-07-17. Enrichment of GO biological process terms was calculated for the DE genes using the goseq R package (version 1.32.0) and GO.db (version 3.6.0). Terms were considered significant (i.e. enriched) at a *p* value threshold of 0.05 after adjustment using the Benjamini–Hochberg false-discovery rate. REVIGO (revigo.irb.hr) [49] was used to summarise GO annotations to sets of differentially expressed genes using a threshold of 0.4 and the SimRel similarity measure.

### Gene cloning and probe synthesis

Gene-specific primers (Additional File 6: Table S5) were designed using Primer3 [177] implemented in Geneious v.8 (Biomatters) against predicted gene model sequences. Parameters were set to produce optimal product sizes of 1–1.5 Kb and to anneal at least 50 bp internally from the ends to increase their efficiency in cases where template cDNAs were not full length. Transcripts were amplified by PCR from cDNAs synthesised from total RNA purified from either Whole Adult or pooled, larval worms. Products were cloned using a StrataClone PCR cloning kit (Agilent Technologies) according to the manufacturer’s instructions, except that all volumes were halved. Transformed plaques were picked and added to 500 μl water, heated to 80 °C to liberate the plasmids, and the resulting mixture used as template for further amplification by PCR using M13 primers. Resulting products (consisting of the gene insert flanked by T3/T7 reverse transcriptase promoter regions and M13 priming sites) were cleaned, quantified and used as templates for probe synthesis. Digoxigenin (DIG)-labelled riboprobes were synthesised from both the sense (+) and anti-sense (−) strands using T3 and T7 reverse transcriptases and DIG-RNA labelling mix (Roche).

### Whole-mount in situ hybridisation

Assays were performed in 1.5-ml Eppendorf tubes that were placed in 50-ml Falcon tubes on a roller to provide continuous agitation during washing steps. A minimum of five worms/assay was used for adults as well as for each larval stage, and separate assays performed for the sense (control) and anti-sense probes. Some assays were repeated two or more times to improve results. PFA-fixed specimens were rehydrated in PBSAT and then permeabilised in 10 μg/ml proteinase K in PBSAT for 10 (adult worms) or 5 (Larvae) mins at room temperature (RT) and then rinsed in 0.1 M triethanolamine pH 7.8 (TEA) followed by 0.25% v/v acetic anhydride in 0.1 M TEA then 0.5% v/v acetic anhydride in 0.1 M TEA, before being washed in PBSAT. They were post-fixed in fresh 4% w/v PFA in PBS for 20 min at RT and thoroughly rinsed in PBSAT. Specimens were equilibrated with hybridisation buffer (50% v/v formamide (Sigma), 5 × saline-sodium citrate buffer (SSC), 100 μg/ml heparin (Sigma), 1× Denhardt’s solution (Sigma), 0.1% v/v Tween 20 (Sigma), 0.1% v/v CHAPS (Sigma), 10 mM EDTA) then prehybridised at 60 °C in hybridisation + buffer, which is hybridisation buffer containing 1 mg/ml yeast RNA (Roche). This was replaced with fresh, pre-warmed hybridisation + buffer containing riboprobe at a concentration of 1 μg/ml and hybridised overnight at 60 °C using a shaking heating block. Probe/hybridisation buffer was removed and stored frozen for future use and the specimens rinsed in pre-warmed hybridisation + buffer to remove unbound probe. Specimens were then washed in 2x SSC followed by 0.2 x SSC buffer (made by dilution in nuclease-free water of 20 × SSC, which is 3 M sodium chloride and 300 mM trisodium citrate in water, pH7), then in 0.1 M maleic acid buffer pH 7.8 (MAB) at RT. This was replaced with blocking buffer consisting of MAB containing 2% w/v bovine serum albumin and 20% v/v heat inactivated lamb serum and incubated for 2 h at RT. This was replaced by fresh solution containing a 1:2000 dilution of anti-DIG antibody coupled to alkaline phosphatase (Roche) and incubated overnight at 4 °C. Antibody solution was removed and the specimens thoroughly washed at RT in MAB followed by alkaline phosphatase buffer. Specimens were transferred to watch glasses, NBT/BCIP (Roche) was added and the specimens left to incubate at RT (or overnight at 4 °C) until a colour reaction was observed. They were then rinsed in PBSAT, post-fixed in 4% w/v PFA in PBS for 1 h, rinsed further in PBSAT and dehydrated in a graded ethanol series. Specimens were cleared in a 1:1 mixture of benzyl alcohol and benzyl benzoate, mounted on microscope slides and stored at 4 °C. WMISH and other protocols can be found on www.olsonlab.com. Mounted specimens were imaged on a Leica DM5000B compound microscope with differential interference contrast and a Leica DFC450C digital camera controlled by Leica Application Suite ver. 4. Images were cropped, and in some cases the overall brightness and/or contrast were digitally enhanced, but no other photo manipulation was made.

## Additional files

**Additional file 1.** Raw and normalised read counts per gene model and sample replicate. **Table S1.1.** RNA-seq sample accessions. **Table S1.2.** RNA-seq mapped read counts by gene model and sample replicate. **Table S1.3.** Normalised (FPKM) mapped read counts by gene model and sample replicate.

**Additional file 2.** Ranked lists of differentially expressed genes for each comparison, showing log2fold changes, mean normalised counts, adjusted *p* values and Gene Ontology terms. **Table S2.1.** Summary of differential expression. **Table S2.2.** Larval cf. Whole Adult. **Table S2.3.** Scolex-Neck cf. Mid. **Table S2.4.** Scolex-Neck cf. End. **Table S2.5.** Mid cf. End. **Table S2.6.** Intersection of up-regulated genes between the Scolex-Neck cf. Mid and Scolex-Neck cf. End contrasts. **Table S2.7.** Intersection of up-regulated genes between the Mid cf. Scolex-Neck and Mid cf. End contrasts. **Table S2.8.** Intersection of up-regulated genes between the End cf. Scolex-Neck and End cf. Mid contrasts.

**Additional file 3.** REVIGO [49] summarised Gene Ontology biological process terms associated with differentially expressed gene models. **Table S3.1.** Larvae cf. Whole Adult. **Table S3.2.** Scolex-Neck cf. Mid. **Table S3.3.** Scolex-Neck cf. End. **Table S3.4.** Mid cf. Scolex-Neck. **Table S3.5.** Mid cf. End. **Table S3.6.** End cf. Scolex-Neck. **Table S3.7.** End cf. Mid.

**Additional file 4: Table S4.** Zinc finger transcription factor genes of interest.

**Additional file 5: Figure S1.** Vitellarium-associated and additional expression foci of the putative zinc finger transcription factor *Hmic*-*zf621400*. **A** Enlarged view of vitellarium expression in mature segments (boxed region of inset). **B–C** Punctate expression is also seen in some specimens, especially in immature segments (arrows show nascent seminal receptacles). **D** Expression by vitelline cells distributed with ova becomes visible in the uterus of mature segments. Abbreviations: esv, external seminal vesicle; gp, genital pore; isv, internal seminal vesicle; sr, seminal receptacle; t, testis; u, uterus; vt, vitellarium. *scale bars* 100 μm (**A, D**), 200 μm (**B, C**).

**Additional file 6: Table S5.** Gene-specific primers and predicted protein sequences of transcripts examined by WMISH.

## Abbreviations

CNS: central nervous system
DE: differentially expressed
FPKM: fragments per kilobase of transcript per million mapped reads
GO: Gene Ontology
WBP: WormBase ParaSite (parasite.wormbase.org)
WMISH: whole-mount in situ hybridisation
