# Supplementary figures and images for: Genome-wide transcriptome profiling and spatial expression analyses identify signals and switches of development in tapeworms
Source: EvoDevo. 2018 Nov 9;9:21. doi: 10.1186/s13227-018-0110-5 (PMC6225667; doi:10.1186/s13227-018-0110-5)

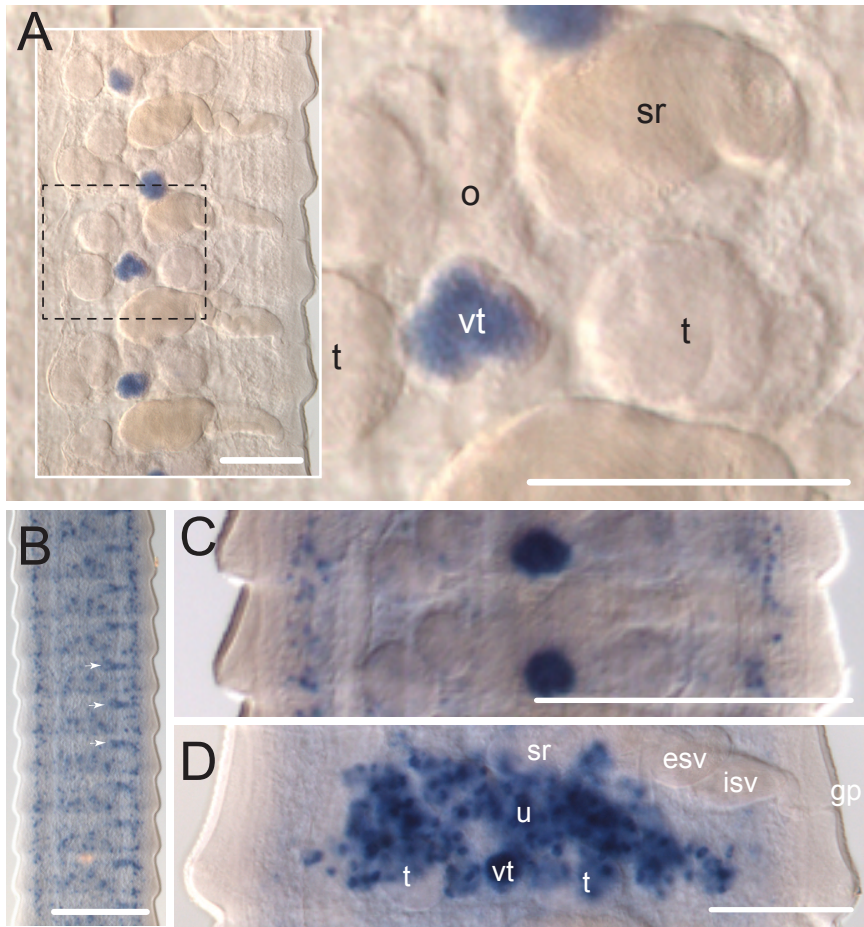

Supplement: Supplementary file 5 — Additional file 5: Figure S1. Vitellarium-associated and additional expression foci of the putative zinc finger transcription factor Hmic-zf621400. A Enlarged view of vitellarium expression in mature segments (boxed region of inset). B–C Punctate expression is also seen in some specimens, especially in immature segments (arrows show nascent seminal receptacles). D Expression by vitelline cells distributed with ova becomes visible in the uterus of mature segments. Abbreviations: esv, external seminal vesicle; gp, genital pore; isv, internal seminal vesicle; sr, seminal receptacle; t, testis; u, uterus; vt, vitellarium. scale bars 100 μm (A, D), 200 μm (B, C). [file 13227_2018_110_MOESM5_ESM.pdf]
